# Reward as a facet of word meaning: Ratings of motivation for 8,601 English words

**DOI:** 10.3758/s13428-025-02762-8

**Published:** 2025-07-29

**Authors:** Doina-Irina Giurgea, Penny M. Pexman, Richard J. Binney

**Affiliations:** 1 Department of Psychology, Bangor University, Gwynedd, Wales UK; 2 Department of Psychology, Western University, London, ON Canada

**Keywords:** Word ratings, Emotion, Drive, Semantic cognition, Lexical decisions

## Abstract

Semantic representations arise from a distillation of multiple sources of information, including sensory, motor, affective, interoceptive, linguistic and cognitive experience. Experience of reward is a highly salient aspect of many human activities, and yet its contribution to semantic processing is not well understood. To address this, the present study took a psycholinguistic approach to measuring and evaluating associations with reward as a facet of word meaning. Behavioural and neurophysiological data suggest that reward processing involves multiple stages and mechanisms. For instance, systems associated with the experience and anticipation of pleasure in response to a reward appear distinct from motivational processes that underlie the pursuit of a stimulus. We sought to collect a novel set of word ratings that capture the full extent of reward-related experience. Initial explorations revealed that reward/pleasure ratings are highly correlated with existing norms of emotional valence. Ratings of association with motivation, however, were only moderately correlated with valence, suggesting they capture distinct semantic information. We therefore conducted a preregistered large-scale study to obtain motivation ratings for 8,601 words. Our analyses suggest these ratings capture aspects of word meaning which are distinct from other semantic dimensions, such as concreteness and valence. Moreover, they explain unique variance in participant performance on lexical, semantic, and recognition memory tasks. We combined motivation and emotional valence ratings to provide a composite measure that might approximate a more general ‘reward’ construct. However, this did not explain additional variance compared to the individual variables. We discuss the implications of these results for neurocognitive theories of semantics.

**Supplementary Information:**

The online version contains supplementary material available at 10.3758/s13428-025-02762-8.

## Introduction

Conceptual knowledge refers to our ‘semantic database’ about the world. It brings meaning to objects, words, events and people, and it is the basis of our ability to interact purposefully with our environment (Patterson et al., 2007). A key challenge for cognitive science, therefore, is to understand how concepts are acquired and represented in the brain. According to *multiple representation* theories, concepts are formed by drawing on multiple, interacting sources of information (Binder & Desai, 2011; Borghi et al., 2019; Connell et al., 2018; Lambon Ralph et al., 2017). Traditionally, there has been a focus on the importance of the sensory-perceptual (e.g., vision, audition) and motor domains, but more recent accounts incorporate a wider range of experiential channels (Barsalou, 2008, 2020). These include linguistic information (Dove, 2018) and other experiences gained through social interaction with others (Borghi et al., 2019; Pexman et al., 2023). Recent research has sought to understand the role of endogenous forms of experience, including interoception (Connell et al., 2018), affect (Kousta et al., 2011; Vigliocco et al., 2014), and introspection/metacognition (Shea, 2018). Behavioural and feature listing studies confirm that these types of information add richness to conceptual representations by, for example, providing additional semantic features or associations (e.g., Corenblum & Pexman, 2025; Diveica et al., 2024b; Harpaintner et al., 2018; Muraki et al., 2022); Villani et al., 2019).

To complement this body of work, in the present study we sought to explore the role of reward processing in semantic cognition, for the following reasons. First, reward is a salient feature of many human activities, such as those involving objects (e.g., food, money) or social interactions (e.g., with friends, lovers), and so it is likely one of the many building blocks of conceptual representations (Binder et al., 2016; Fennell & Baddeley, 2013). Second, the pursuit and receipt of reward greatly influences the types and frequency of experiences we engage in. Therefore, it could shape the way in which we learn the meaning of things (Hughes & Zaki, 2015; Madan, 2017). The idea that there is a coupling of reward processing and semantic cognition is consistent with the observation that disturbances to stimulus–reward associations contribute to profound social-semantic impairments in certain neurological disorders (i.e., Frontotemporal Dementia; for a related review, see Rankin, 2020; Rouse et al., 2024). Indeed, it is possible that hedonic response and reward valuation are particularly important for the acquisition of certain types of concepts, like object concepts or socially relevant concepts (Pexman et al., 2023), or in the development of personal semantics (Arioli et al., 2021; Renoult et al., 2016). Despite this, reward processing is seldom discussed within contemporary theories of the cognitive underpinnings of semantics. In an initial attempt to address this gap, in the present study we took a psycholinguistic approach, obtaining a novel set of normative ratings aimed at quantifying reward experience as a facet of word meaning. We then evaluated the extent to which these new ratings capture word meaning uniquely from other semantic variables, including concreteness and emotional valence. Finally, using item-wise hierarchical regression analyses, we found that the new ratings explain unique variance in participant performance on lexical-semantic tasks. Demonstrating the behavioural relevance of a semantic variable is requisite for its inclusion in psychological theory.

Reward has been characterized as the experience of satisfaction and enjoyment that follows a state of wanting which compelled us to engage in a certain behaviour (Matyjek et al., 2020). As mentioned, emotional attributes of concepts, like pleasure, have been shown to influence semantic processing. For example, Kousta et al. (2009) found that words with affective associations were processed faster than neutral words in a lexical decision task (also see Kousta et al., 2011; Vigliocco et al., 2014; Warriner et al., 2013). However, it was not only positive associations that facilitated processing, but also negative associations. Indeed, there are behavioural and neurophysiological data from both humans and animals which elucidate how reward processing is more complex and multifaceted than the formation of simple hedonic associations (Schultz, 2015). For example, one influential theory argues that the brain’s reward system comprises dissociable components (Berridge & Robinson, 2016; Robinson & Berridge, 1993; Schultz, 2015). The first mechanism, *liking,* is that emotional aspect, the pleasurable impact of receiving a reward. The second is *wanting* (or incentive salience), a form of motivation which determines how much an individual prefers and pursues the reward. Animal lesion studies suggest that liking and wanting can be selectively abolished (Berridge et al., 1989; Wyvell & Berridge, 2000), although both liking and wanting are necessary for full reward (Berridge & Kringelbach, 2015). Moreover, the potential independence of wanting and liking makes it theoretically possible that individuals could mobilize effort to obtain an outcome that they do not expect to like, or is devoid of pleasure (Berridge & Aldridge, 2008). In the human experimental literature, this distinction is controversial (Havermans, 2011; Pool et al., 2016), and alternative distinctions have been suggested including that between (a) expected pleasantness, which involves high-level predictive cognition and memories of past liking, and (b) affective relevance which is derived from an interaction between the stimulus attributes and the motivational state of an individual (see Pool et al., 2016, for a review). Those investigating reward and affect processing in humans recognize that pleasure can take a multitude of forms including the physical, the intellectual and the social as well as emotional pleasure (Dubé & Le Bel, 2003). The literature also highlights a larger variety of motives than that typically considered in the animal literature, including those related to social dynamics (Sander et al., 2005). To better understand the role of reward processing in meaning representation, research needs to move beyond the role of affect processing alone. To this end, we sought to employ an inclusive definition of reward-related experience that could capture greater complexity.

As a starting point, we revisited an influential study by Binder et al. (2016) which examined a novel set of 65 experience-based attributes from the sensory, motor, affective, and cognitive domains and their contributions to the meaning representation of 535 words. Of particular interest to us was a factor analysis applied to the ratings of 496 words, including nouns and verbs (*N* = 62), which yielded a latent variable that the authors labelled ‘reward’. The three highest-loading attributes on the reward factor were ‘benefit’ (‘something or someone that can help you or others’), ‘needs’ (‘something or someone you could not live without’), and ‘drive’ (‘something or someone that makes you do something’). The first two attributes may have captured the reward value of a concept, whereas, according to Binder and colleagues, the term ‘drive’ is synonymous with ‘reward prediction’ and ‘reward anticipation’, and it may capture general motivation associated with a concept. Purposefully or not, the attributes that loaded on ‘reward’ appear to mirror the distinctions made in the affective neuroscience literature. Interestingly, though, the reward factor was distinct from two other factors that captured positive and negative valence, and this could suggest it reflects reward-related processes that go beyond affective experiences.

Binder et al.’s (2016) model was able to distinguish a priori conceptual categories (e.g., artefacts, living things and abstract entities) and capture semantic similarity, and, in doing so, highlights the potential role of reward and drive states in meaning representation. They did not, however, explore whether the attributes or latent variables explain variance in participant lexical-semantic task performance, which is a gold standard for psychological theory. To establish a proof of principle, we conducted a preliminary analysis using item-wise hierarchical regression to examine whether Binder et al.’s word ratings on the benefit, needs and drive attributes are related to behaviour in a lexical decision task (LDT; Balota et al., 2007) and in the English Crowdsourcing Project (ECP) word knowledge task (Mandera et al., 2020). We included lexical and semantic predictors (letter length, frequency (Brysbaert & New, 2009), rating-based age of acquisition (Kuperman et al., 2012), concreteness (Brysbaert et al., 2014), valence (Warriner et al., 2013) and semantic diversity (Hoffman et al., 2013) as factors that are known to predict behaviour in lexical tasks. These acted as controls that could isolate the unique relationships of reward attributes to reaction times (RTs) and error rates. Because we wanted values for all these variables, we were limited for this preliminary analysis to a word sample of 396 items. The full details can be found in the supplementary information (Section S1 of supplementary materials) but, in brief, while we did not find an effect of the individual variables (benefit, needs, drive), we did find an effect of a composite computed from factor scores. This was specific to predicting behaviour in the ECP word knowledge task (1% variance explained), where words with high scores on the composite reward variable tended to have faster RTs. We therefore obtained preliminary evidence that ratings of reward attributes are related to semantic processing. Somewhat complementary findings have been obtained by Wurm (2007), who found that word ratings of danger and usefulness (which are motivational attributes) relate to RTs in an auditory lexical decision task. Moreover, Amsel et al. (2012) found that latent semantic variables pertaining to enhancing chances of survival (named ‘avoiding death’ and ‘locating nourishment’) accounted for significant variance in lexical and semantic decision latencies. However, these studies and our preliminary analysis using Binder et al.’s (2016) ratings were based on small word samples, which constrains the conclusions that can be drawn, and it is possible the findings do not generalize to other sets of words (for example, those with a greater number of abstract words, verbs, etc). In the present study, we obtained ratings of reward-related experience for a large set of English words constituting the most common parts of speech. To facilitate future research endeavours, we ensured item overlap of our word sample with behavioural mega-studies and other theoretically important psycholinguistic dimensions. This also enabled us to conduct an initial analysis of the relationships between the new measure and other semantic variables, as well as task performance.

Our intent was that the ratings captured the full complexity of reward experience (including non-affective forms of pleasure/satisfaction), and so we took an inclusive approach to the definitions. We also wished to capture the distinctions made in the affective neuroscience literature between liking/expected pleasantness and motivational systems. To this end, we conducted an initial exploration that piloted two separate measures that we named ‘pleasure’ and ‘motivation’ (see Supplementary Materials S2 and S3 for further details regarding the stimuli, ratings definitions, participant instructions, sampling, procedure, and analysis). For both measures, we obtained 20 ratings for each of 60 words. The word sample was selected with respect to the definitions and to span the expected continuum of both ‘pleasure’ and ‘motivation’. The full results of the pilot can be found in the supplementary information 2, but the key findings were as follows. First, ratings of a word’s association with pleasure were strongly and positively correlated with pre-existing measures of emotional valence (*r* =.93; Warriner et al., 2013). Thus, despite attempting to capture other forms of pleasure, we appeared to measure the same emotion construct. The ratings of a word’s association with motivation, on the other hand, were only moderately correlated with emotional valence (*r* =.56). This suggests that this measure is capturing different reward-related attributes, and possibly something akin to Binder et al.’s (2016) ‘drive’ attribute. This led to the decision that, in the full study reported below, we should focus on norming words for their association with motivation. The pleasure component of reward processing would likely be adequately captured by Warriner et al.’s (2013) emotion-related ratings, and we aimed to explore combining these with the new measure to create a resultant composite measure of reward.

In summary, the aims of the present study were as follows:

1. Collect motivation ratings for a large set of English words to provide a useful resource for future research endeavours;
2. Explore to what extent these new ratings capture aspects of word meaning that are distinct from those measured via other related semantic variables, such as concreteness and emotional valence;
3. Test whether motivation ratings can explain variance in performance on lexical and semantic tasks; and
4. Evaluate whether a composite measure of motivation and emotional valence can be used to quantify a more general ‘reward’ construct.

## Methods

The study plans and analysis plans were preregistered (https://osf.io/jwhbs).

### Participants

Participants were recruited online using Prolific (2022; https://www.prolific.com). All participants self-reported as being fluent English speakers with no language disorders. The total number of participants was 649 (413 male, 232 female, 4 unspecified, *M*_age_ = 35.16, *SD*_age_ = 12.14). The average completion time was 35 min, and participants were compensated £4. The final sample included 601 participants, with ages ranging from 18 to 77 years (*M*_age_ = 34.99; *SD*_age_ = 11.94; 379 male, 218 female, 4 unspecified). English was the first language for 400 (66.56%) participants. Of the remaining 201 (33.44%) participants, 97 self-reported being proficient in English, 84 advanced and 20 intermediate. A total of 267 (44.43%) participants were monolingual, while the remaining 334 (55.57%) reported speaking more than one language.

### Stimuli

We collected ratings for 8,765 words. These included 5,431 nouns, 1,939 adjectives, 1,356 verbs, 6 adverbs, and 16 other parts of speech (Brysbaert et al., 2012). This information was not available for 16 words. The stimulus set was initially curated on the basis of being words previously rated for concreteness (Brysbaert et al., 2014), emotion dimensions (valence, arousal, dominance; Warriner et al., 2013) and, most recently, socialness (*N* = 8,388; Diveica et al., 2023), and which span the entire continuum of these dimensions. A large majority of these words have also been rated on other semantic dimensions, including those comprising the Lancaster Sensorimotor Norms (Lynott et al., 2019) and word association norms (De Deyne et al., 2019), amongst others (Brysbaert et al., 2019; Scott et al., 2019). Moreover, this initial word list was also chosen as it has significant overlap with those used in several behavioural mega-studies (i.e., the Calgary Semantic Decision Project by Pexman et al., 2017, but also Balota et al., 2007, and Mandera et al., 2020), which allowed us to evaluate the relationships between the ratings and task performance. We also included a further 377 words for which socialness ratings had been acquired after the original study by Diveica et al. (2023). This was a minor deviation from the pre-registration of the present study. Valence norms were not available for 93 of these extra words, and concreteness norms are missing for 43. These words were mostly verbs in the past tense and infinitives. We used 60 of the 8,765 words as a set of control items which were presented to every participant and used during the data cleaning process (see below). These words were also used in a pilot study (*N* = 20 participants) to check the participants’ understanding of the task instructions and to obtain an initial assessment of the reliability of ratings (see Supplementary Materials S2). They were selected to vary in concreteness (Brysbaert et al., 2014), emotional valence ratings (Warriner et al., 2013), and in the mean pilot motivation ratings.

We used an additional 12 words as practice words to be rated before the main rating task, so that participants could familiarize themselves with the task requirements. These words were also used as practice words by Diveica et al. (2023) and were not part of the main stimulus set in that study. The practice words varied in concreteness (Brybaert et al., 2014) and emotional valence (Warriner et al., 2013).

The 8,705 words (excluding control words) were split into two lists (4,352 words and 4,353 words) to satisfy pragmatic constraints in Qualtrics (2024). The lists were matched for mean letter length, frequency (log subtitle frequency; Brysbaert & New, 2009), concreteness (Brysbaert et al., 2014), and emotional valence (Warriner et al., 2013).

### Procedure

The words were presented using Qualtrics (2024) and accessed by participants via the Prolific online recruitment platform (www.prolific.com). The second list was made available as a separate study after collecting at least 25 ratings per word for all words in the first list. We ensured that participants were precluded from rating both lists. All participants first provided informed consent, demographics, and read the instructions of the word rating task. Subsequently, they rated the 12 practice words and were then able to read the instructions again if they wished to do so. They then rated the main set of stimuli. Each participant rated 340 words randomly selected from one of the two lists, plus the 60 control words. The control words were intermixed with the other items.

The full instructions given to participants are available in Section S4 of the supplementary materials. Briefly, the participants were asked to rate the degree to which each word’s meaning was associated with motivation, which was described as ‘a sense of desire to achieve or obtain a goal’. Further, it was specified that a word could be related to motivation regardless of whether it refers to something positive or negative, because even aversive stimuli (e.g., a tornado) can motivate a decision or action.

Participants were presented with 25 words per page and were provided with a brief reminder of the task at the top of each page: ‘Instructions summary: Rate the degree to which the word's meaning is related to motivation’. Ratings were provided on a seven-point Likert scale presented horizontally below each word, with 1 representing a low motivation rating and 7 representing a high motivation rating. There was an additional option to indicate that they did not know the word (‘I don’t know the meaning of this word’). Data were collected until we obtained at least 25 ratings per word.

### Data cleaning

In total, we collected 260,034 observations. The data cleaning process implemented methods used in previous studies to identify careless or insufficient effort in responding (Curran, 2016) and computer-generated random responses (Dupuis et al., 2019). We also used a set of procedures developed for previous large-scale word-rating studies to maximize the reliability of ratings, such as looking for consecutive identical responses and checking the correlations between individual scores and mean rating scores (Brysbaert et al., 2014; Diveica et al., 2023; Muraki et al., 2023; Pexman et al., 2019). Data cleaning steps are described next.

First, we removed data from participants who responded with ‘I don’t know the meaning of this word’ for more than 25% of items (*n* = 1) and/or provided the same rating more than 25 times in a row (*n* = 31). We also removed all data for participants who completed less than 33% of the task (*n* = 0). Next, we examined each participant's ratings of the control words and computed the correlation with the mean ratings of those words in the pilot study. We removed data from 16 participants with a correlation coefficient less than. 20. We also computed correlations between each participant’s ratings of the control words and the mean ratings of all other participants and removed their data if we observed a correlation coefficient of less than .10 (*n* = 0). Finally, we removed any words reported as not known by more than 15% of raters from all further analyses; 164 words were excluded by this criterion.

The final dataset contained 8,601 words and 237,076 observations, of which 3,040 were ‘I don’t know the meaning of this word’ responses. Words in the final dataset had 23.22 valid ratings on average (*SD* = 2.04), ranging from 16 to 32 ratings. Following data cleaning, out of the final 8,601 words, 8,358 (97.17%) words had at least 20 valid ratings. Corenblum and Pexman (2025) found that mean ratings of cognition stabilized at 20 ratings per word, with minimal changes in standard deviation.

### Data analysis

All data cleaning, analyses, and visualizations were performed in RStudio (version 4.3.3; 2024). Our data cleaning pipeline and analytical plans were pre-registered (https://osf.io/jwhbs) and will be described in more detail below.

## Results

### Descriptives

**Fig. 1:**
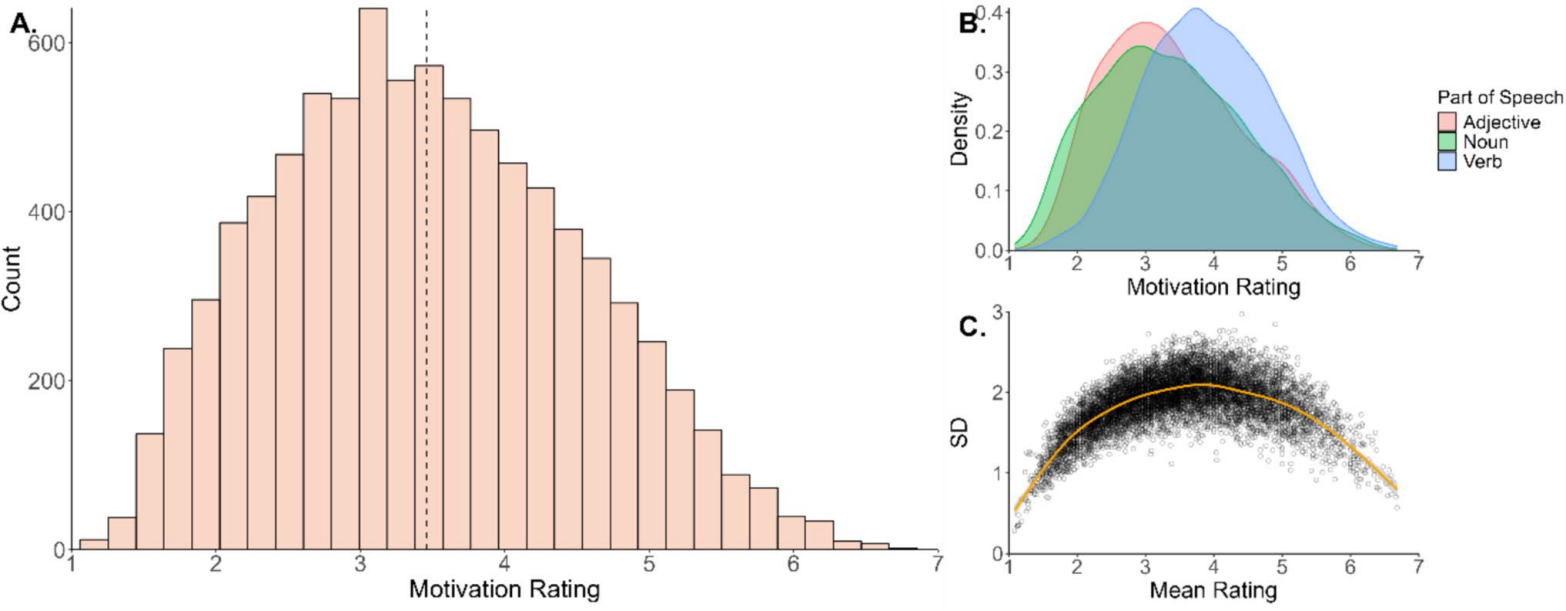
Distribution of motivation ratings. **A** Histogram of motivation ratings for the 8,601 words; the dotted line represents the mean. **B** Density plots displayed as a function of part of speech. **C** Standard deviation of the motivation ratings plotted against their respective mean rating, along with a loess line (in orange) that highlights the relationship

All raw data and the mean item-wise motivation ratings are available on the Open Science Framework (OSF) project page (available at https://osf.io/3vx9y/). The motivation ratings overall had a unimodal distribution (*M* = 3.46; *SD* = 1.05; Fig. 1A), and this was true for each of the most common parts of speech (Fig. 1B). Additional descriptive statistics are presented in Table 1. The average standard deviation of the ratings was 1.87 (*SD* = 0.33), and participants provided more consistent responses at the extremes of the scale (Fig. 1C). Examples of words at the extremes of the motivation dimension are given in Table 2. Words like ‘aspiration’, ‘ambition’ and ‘win’ received high motivation ratings, while words like ‘beige’, ‘bottle’ and ‘goose’ received low motivation ratings, suggesting good face validity.

**Table 1:** Descriptive statistics of the mean motivation ratings for 8,601 words and for each part of speech

Descriptive statistic	Mean ratings	Adjective	Adverb	Name	Noun	Number	Preposition	Pronoun	Verb	Other
Mean	3.46	3.41	4.23	2.55	3.36	2.09	5.04	3.80	3.92	3.89
Median	3.39	3.29	4.53	2.46	3.29	1.94	5.04	3.80	3.87	3.92
Standard deviation	1.05	1.00	0.98	0.80	1.07	0.81	NA	NA	0.91	0.54
Minimum	1.08	1.08	2.64	1.45	1.12	1.33	5.04	3.80	1.47	3.04
Maximum	6.68	6.54	5.37	3.96	6.68	4.75	5.04	3.80	6.59	4.68
1 st Quartile	2.65	2.62	3.77	1.81	2.55	1.76	5.04	3.80	3.25	3.41
3rd Quartile	4.20	4.09	4.74	3.22	4.12	2.16	5.04	3.80	4.57	4.37
Skewness	0.27	0.43	−0.47	0.23	0.33	2.43	NA	NA	0.13	−0.06
Kurtosis	−0.53	−0.50	−1.48	−1.46	−0.50	5.40	NA	NA	−0.34	−1.52
*N*	8,601	1,912	6	15	5,300	14	1	1	1,336	16

**Table 2:** Example words at the extremes of the motivation dimension

Highest-rated words	Rating	Lowest-rated words	Rating
aspiration	6.68	beige	1.08
ambition	6.67	bottle	1.12
win	6.59	goose	1.12
achieve	6.59	hertz	1.14
success	6.58	bowl	1.14
motivate	6.57	shoelace	1.14
ambitious	6.54	otter	1.14
accomplish	6.52	pelican	1.19
career	6.48	sawmill	1.19
bravery	6.43	jaw	1.24
freedom	6.40	pillowcase	1.24
encourage	6.40	apricot	1.25
perfectionist	6.38	tumbleweed	1.26
profit	6.38	east	1.26
goal	6.36	rectangular	1.26
conquer	6.35	cauliflower	1.28
desire	6.35	walrus	1.29
triumph	6.32	bellman	1.29
growth	6.32	raisin	1.29
aspire	6.27	albino	1.30
energetic	6.26	cornbread	1.30
willpower	6.26	coleslaw	1.32
inspire	6.25	driftwood	1.32
health	6.24	lettuce	1.32
promote	6.22	radish	1.32

### Reliability and validity

We used the one-way intra-class correlation coefficient (ICC), with variances estimated via a random effects model with a global intercept and a random intercept per word (Brysbaert, 2019; Brysbaert & Stevens, 2018), and Spearman–Brown corrected split-half reliability (using 100 random splits; splithalf.r function in multicon package version 1.5). We checked the validity of the ratings for the 60 control words by computing the correlations between the ratings obtained in the pilot study and those obtained in the main study. We also computed the correlation between the new motivation ratings and ratings on the ‘drive’ dimension reported by Binder et al. (2016), although the datasets only overlapped on 529 items.

We observed good reliability for the mean motivation ratings, as indicated by an ICC of 0.88. The mean Spearman–Brown corrected split-half reliability was 0.997 (*SD* = 0.13) across 100 random splits, suggesting high reliability across ratings of the control items (the 60 items rated by all participants). The new motivation ratings of control words were strongly and positively correlated with the ratings collected in the pilot study (*r* = 0.91). Ratings of 529 words were strongly correlated with the ‘drive’ ratings collected by Binder et al. (2016; *r* = 0.73), suggesting good validity.

### Zero-order correlations

**Fig. 2:**
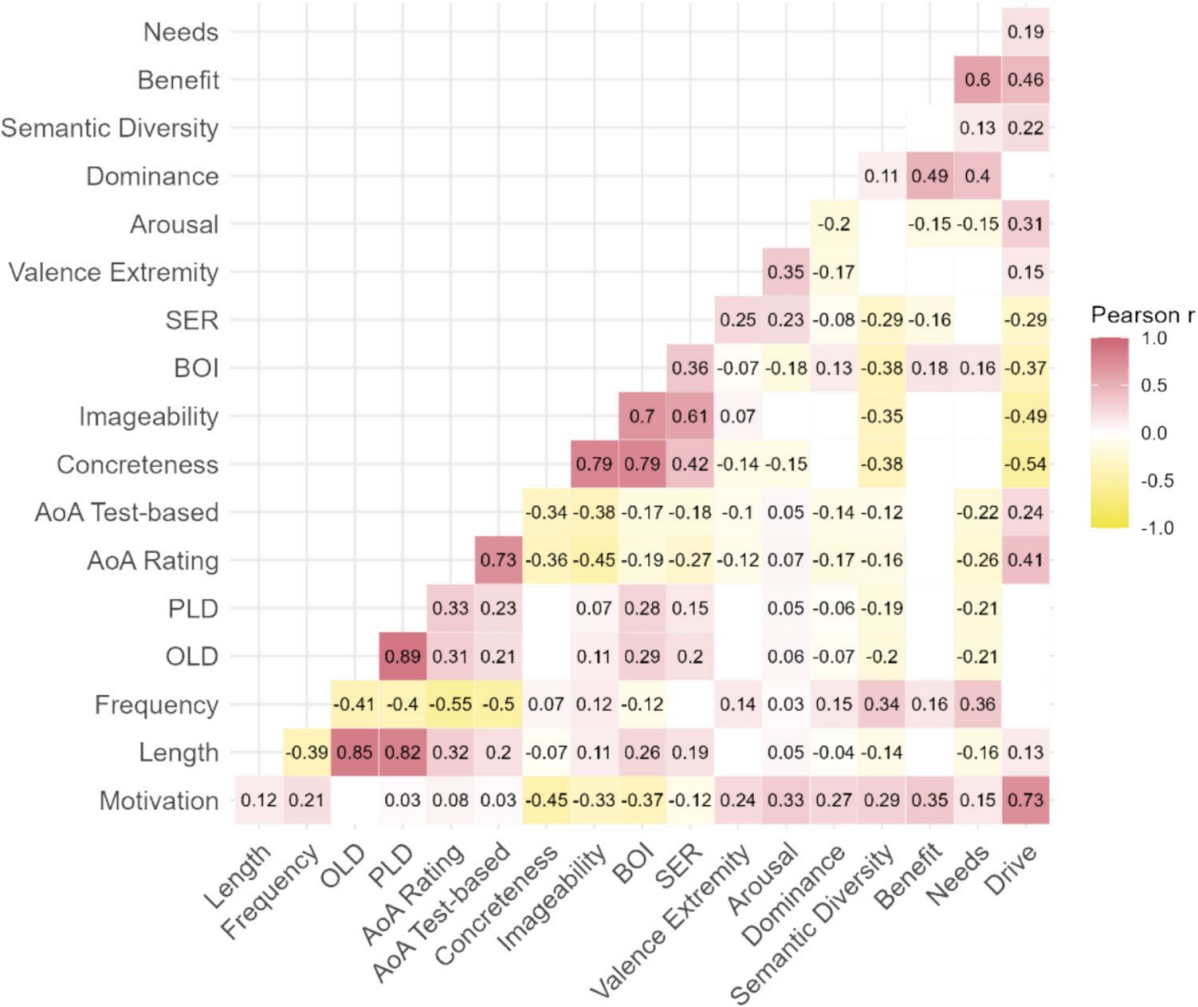
Correlations between mean motivation ratings and all variables of interest, including semantic and lexical variables. Only the correlations significant at *p* <.01 are displayed. The colours and numerical values indicate the strength and direction of the relationships. The numbers of items for each variable were: 8,601 (length), 8,561 (concreteness), 8,509 (valence, valence extremity, arousal, dominance), 8,493 (AoA rating), 8,400 (frequency), 8,263 (OLD, PLD), 7,502 (AoA test), 7,340 (semantic diversity), 4,232 (BOI), 2,878 (imageability), 2,830 (SER), 529 (benefit, needs, drive). SER = sensory experience rating; BOI = body–object interaction; AoA = age of acquisition; PLD = phonologic Levenshtein distance; OLD = orthographic Levenshtein distance

We examined the zero-order correlations between the motivation ratings and various lexical and semantic properties of the words, as well as the reward-related ratings (benefit, needs, drive) collected by Binder et al. (2016; see Fig. 2). This could help characterize our measure of motivation. The lexical variables included letter length, orthographic Levensthein distance (Yarkoni et al., 2008), phonological Levensthein distance and frequency (log subtitle frequency; Brysbaert & New, 2009). To assess the relationships between the motivation ratings and sensorimotor information, the semantic variables included concreteness (the degree to which the word’s referent can be experienced through one of the five senses; 1= abstract; 5 = concrete; Brysbaert et al., 2014), body–object interaction (BOI; the ease with which the human body interacts with a word’s referent; 1 = low body–object interaction; 7 = high body–object interaction; Pexman et al., 2019), imageability (the ease with which a word arouses a mental image of the referent; Cortese & Fugett, 2004; Schock et al., 2012), and sensorimotor experience ratings (SER; the extent of evoked sensory/perceptual experience; 1 = low sensory experience; 7 = high sensory experience; Juhasz & Yap, 2013). To assess the relationship between the motivation ratings and affective experience, which could equate to the pleasure component of reward processing, the semantic variables included Warriner et al.’s (2013) ratings of emotional valence (i.e., a word’s meaning as negative, neutral or positive; 1 = negative/unhappy; 9 = positive/happy), valence extremity (i.e., the absolute value of the difference between the valence rating from 5, which was the neutral point on the scale, representing word meaning as neutral or emotional), arousal (the degree to which the word evokes feelings of excitement as opposed to calm; 1 = excited; 9 = calm), and dominance (the degree to which the word evokes feeling of being controlled or being in control; 1 = controlled; 9 = in control). Additionally, to assess the relationships between the motivation ratings and measures of linguistic experience, the semantic variables included semantic diversity (the extent to which a word appears in semantically diverse contexts; Hoffman et al., 2013), rating-based age of acquisition (AoA; Kuperman et al., 2012), and a test-based AoA measure (Brysbaert & Biemiller, 2017; Dale & O’Rourke, 1981). See section S6 of the supplementary materials for scatterplots of the relationships between motivation and other psycholinguistic dimensions.

The new motivation ratings were negatively correlated with concreteness (*r* = −0.45), imageability (*r* = −0.33), BOI (*r* = −0.37), and SER (*r* = −0.12), which suggests that words strongly associated with motivation are associated with less sensorimotor information. In contrast, the motivation ratings had a positive correlation with valence extremity (*r* = 0.24), arousal (*r* = 0.33), and dominance (*r* = 0.27), suggesting that words strongly associated with motivation tend to have more associated affective information. There was also a positive correlation with semantic diversity (*r = *0.29), suggesting that words highly associated with motivation tend to appear in more semantically diverse contexts. The motivation measure had a positive correlation with Binder et al.’s (2016) ‘benefit’ measure, but it was most strongly correlated with the ‘drive’ measure. In Section 5, Table S1 of the supplementary materials, we report zero-order correlations of all lexical/semantic variables with the behavioural dependent variables, but these relationships were explored in greater depth via the regression analyses.

### Hierarchical regression analyses

We conducted a series of pre-registered hierarchical regression analyses (using the lm function in the stats package; version 3.6.2) to explore the relationships between motivation and behavioural performance on lexical and semantic tasks, including the lexical decision task (LDT) from the English Lexicon Project (ELP; Balota et al., 2007), the English Crowdsourcing Project (ECP) word knowledge task (Mandera et al., 2020), the semantic decision task (SDT) from the Calgary Semantic Decision Project (Pexman et al., 2017) and the recognition memory task (RMT; Cortese et al., 2010, 2015). The outcome variables were RT and error rates on the LDT and SDT, which require participants to indicate whether a stimulus is a word or a non-word, and to indicate whether a word has an abstract or concrete meaning, respectively. For SDT performance, we analysed concrete and abstract judgements separately. This was motivated by previous research showing that different semantic variables explain concrete and abstract decisions (e.g., concrete judgements are facilitated primarily by concreteness, while abstract judgements are facilitated by higher emotional valence and semantic diversity; Diveica et al., 2024b; Newcombe et al., 2012; Pexman & Yap, 2018). This distinction was made based on the labelling in the original study (Pexman et al., 2017). In addition, RT and word prevalence were used as outcome variables for the ECP word recognition task, which requires participants to indicate whether they know a word. The RMT requires participants to read a list of words and later recognize these words in a new list where they are intermixed with previously unseen words. We used as outcome variables the proportion of hits (items correctly recognized as seen prior to test; ‘old’), false alarms (items incorrectly recognized as seen prior to test; ‘new’), and the difference between the rate of hits and false alarms (which can be interpreted as an index of memory accuracy).

Collectively, these tasks involve both shallow processing (LDT, ECP) and deeper semantic processing (SDT, RMT; Muraki et al., 2022), and the extent to which semantic variables can explain variance differs between tasks, with the amount in LDT and ECP being substantially smaller than in the SDT (Heard et al., 2019; Taikh et al., 2015; Yap et al., 2012). This range of tasks allowed us to explore the effects of our motivation ratings across a broad set of task contexts, taking into account the dynamic and context-dependent nature of conceptual representations (Pexman, 2020; Yee & Thompson-Schill, 2016).

The variables used in the analyses were introduced in each model in two steps as either (1) control variables, including word length, frequency (log transformed SUBTLEX; Brysbaert & New, 2009), and AoA (Kuperman et al., 2012), or (2) semantic variables, including concreteness (Brysbaert et al., 2014), valence extremity (Warriner et al., 2013), semantic diversity (Hoffman et al., 2013), and our ratings of motivation. This approach ensured that relevant lexical and semantic variables were controlled for (Winter, 2019) while assessing the predictive value of motivation ratings. Valence extremity was chosen a priori, as opposed to dominance and arousal, to be consistent with our prior norming study (Diveica et al, 2023). However, given the zero-order correlations we observed between the other affect measures and the motivation measure, we performed additional ad hoc analyses that included arousal and dominance as predictors. As can be seen in Section S8, the inclusion of these predictors did not change the overall results. In the case of models predicting the outcomes from the RMT, we included a further control lexical variable, orthographic Levensthein distance (OLD; the number of insertions, deletions, and substitutions needed to transform one word into another; near 1.0 suggests that the word is very similar to its 20 neighbours; far from 1.0 suggests that the word is orthographically unique; Yarkoni et al., 2008), in the first step. This was based on its established effects on memory, such as better memory for more orthographically unique words (Cortese & Fugett, 2004). We mean-centred all the predictors, and we used z-scored RTs to reduce the impact of individual differences in processing speed (Faust et al., 1999).

#### Performance on lexical tasks

**Fig. 3:**
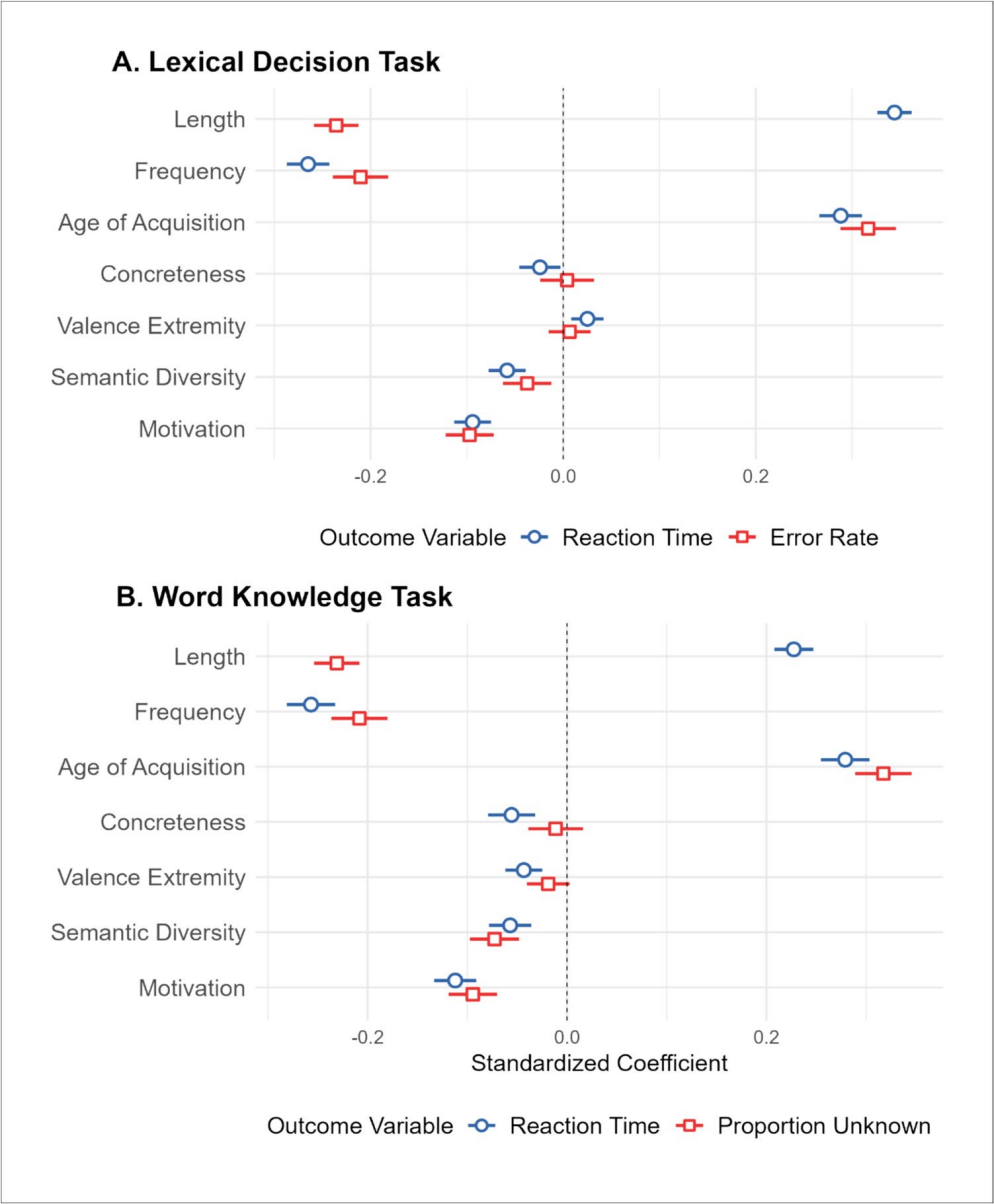
Plot illustrating the standardized coefficient weights and 95% CIs for the second step of the hierarchical regression analyses predicting the lexical task outcome variables. **A** Standardized beta coefficients for LDT RTs (blue) and errors (red). **B** Standardized beta coefficients for ECP word knowledge task RTs (blue) and proportion of people reporting not knowing a word (red)

We examined the relationship between motivation and lexical-semantic processing using behavioural responses from the ELP LDT (Balota et al., 2007) and the ECP word knowledge task (Mandera et al., 2020). There were 7,127 items for which we had data for all variables of interest in the analysis predicting LDT performance. These were subject to item-wise hierarchical regression analyses. In the first step, we entered the control variables and observed that they were all significant predictors of LDT latencies, with faster RTs being associated with words that are shorter, more frequent, and acquired earlier. The addition of the semantic variables (including motivation) significantly improved the model fit, explaining an additional 1% of the variance. All the semantic variables were significant predictors of LDT RTs, with faster reaction times for words that are more concrete, less extremely valenced, present in more semantically diverse contexts, and more strongly associated with motivation. When predicting LDT error rates, all control variables were significant predictors, with fewer errors for words that were longer, more frequent, and acquired earlier. The addition of the semantic variables significantly improved the model fit, explaining an additional 1.1% of variance. However, only semantic diversity and motivation were significant predictors, with fewer errors for words present in more semantically diverse contexts and that are highly associated with motivation (see Fig. 3A for illustrated standardized coefficients; see Section 5 of supplementary materials for all regression tables).

There were 7,210 items for which we had data for all variables of interest in the analysis predicting performance on the ECP word knowledge task. When predicting RTs, all control variables were significant predictors, with faster reaction times for shorter, more frequent, and early-acquired words. The addition of the semantic variables significantly improved the model fit, explaining an additional 1.5% of variance. All semantic variables were significant predictors, with faster RTs for words that were more concrete, more extremely valenced, more semantically diverse, and more strongly associated with motivation. In explaining the proportion of participants reporting not knowing a word, all control variables were significant predictors, with less prevalent words being shorter, less frequent, and acquired later. The inclusion of the semantic variables significantly improved the model fit, explaining an additional 1.5% of variance. Here, only semantic diversity and motivation were significant predictors, with words that were less semantically diverse and less strongly associated with motivation reported as unknown by more participants (see Fig. 3B for illustrated standardized coefficients; and Section S5 of the supplementary materials for additional detail).

#### Semantic decision task

**Fig. 4:**
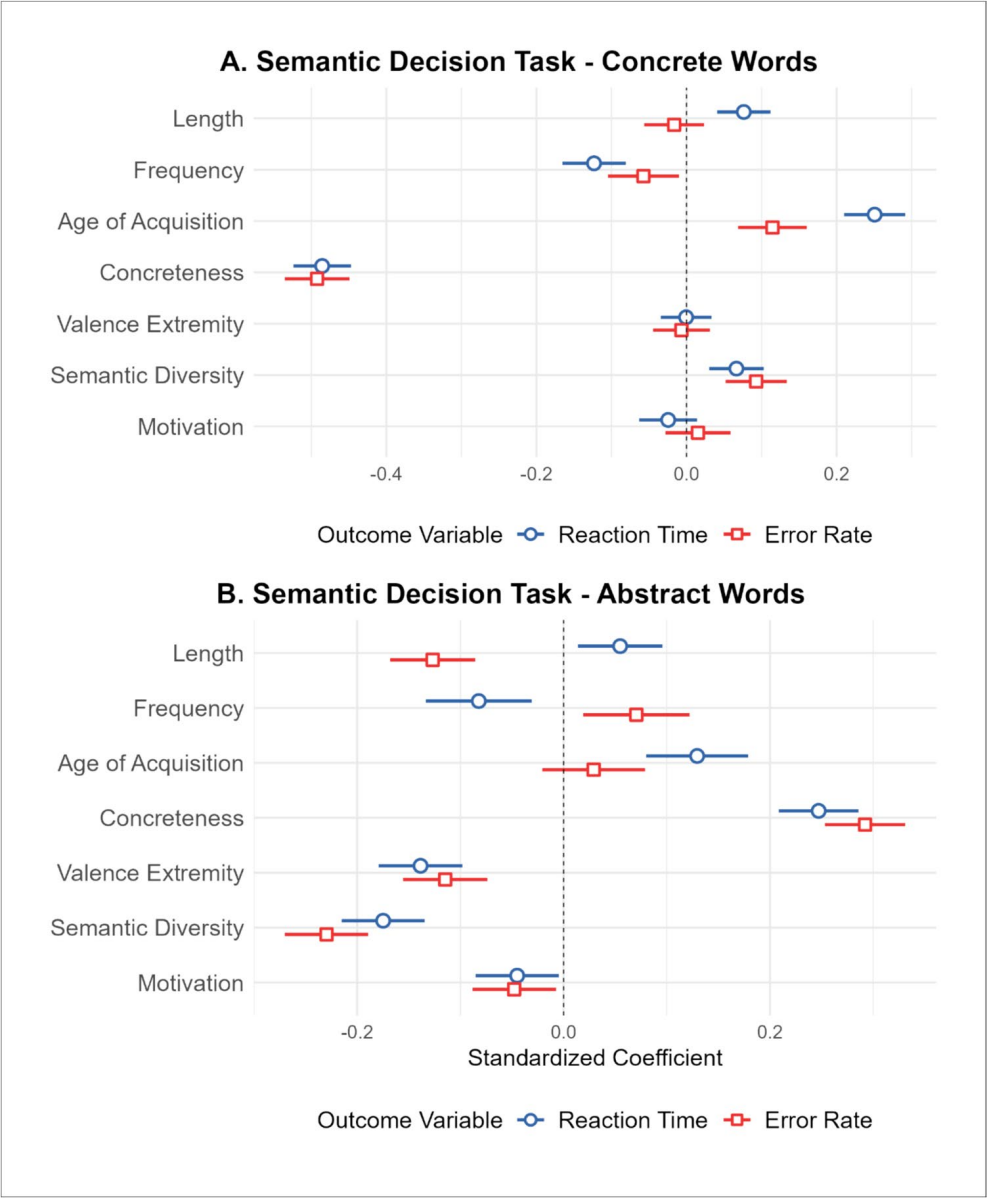
Plot illustrating the standardized coefficient weights and 95% CIs for the second step of the hierarchical regression analyses predicting the semantic decision task outcome variables. **A** Standardized beta coefficients for concrete SDT RTs (blue) and errors (red). **B** Standardized beta coefficients for abstract SDT RTs (blue) and errors (red)

We examined the relationship between motivation and responses on the SDT (Pexman et al., 2017) for concrete and, separately, abstract words. There were 1,958 concrete words with values for all variables of interest. All control variables were significant predictors of RTs, with faster RTs for shorter, more frequent, and early-acquired words. Adding the semantic variables significantly improved the model fit, explaining an additional 20.8% of variance. Only concreteness and semantic diversity were significant predictors, with faster reaction times for more concrete and less semantically diverse words. For the error rates, only AoA was a significant predictor among the control variables, with fewer errors for early-acquired words. The inclusion of the semantic variables significantly improved the model fit, explaining an additional 23.4% of variance in response errors. Only concreteness and semantic diversity were significant predictors, with fewer errors for more concrete and less semantically diverse words (see Fig. 4A for illustrated standardized coefficients).

There were 2,150 abstract words with values for all variables of interest. Of the control variables, only frequency and AoA were significant predictors of RTs, with faster reaction times for more frequent and early acquired words. The addition of the semantic variables significantly improved the model fit, explaining an additional 11.5% of variance. All semantic variables were significant predictors, with faster RTs for words that were less concrete, more extremely valenced, more semantically diverse, and more strongly associated with motivation. Only length was a significant lexical predictor of SDT error rates, where fewer errors were associated with longer words. The addition of the semantic variables significantly improved the model fit, explaining an additional 15.9% of variance. All semantic variables were significant predictors, with fewer errors for words that were less concrete, more extremely valenced, more semantically diverse, and more strongly associated with motivation (see Fig. 4B for illustrated standardized coefficients).

#### Recognition memory task

**Fig. 5:**
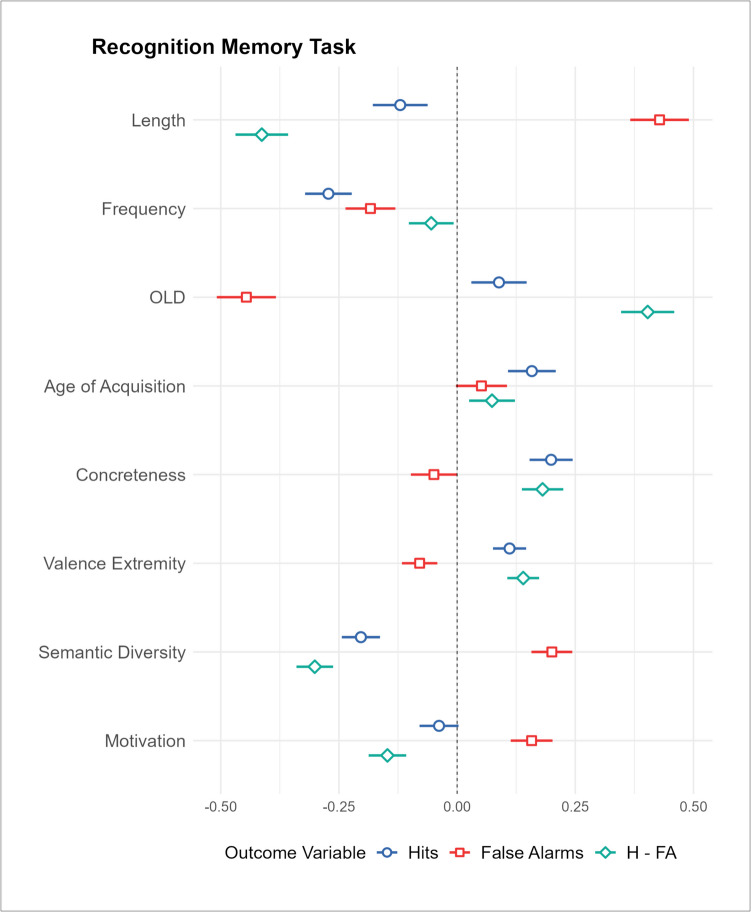
Plot illustrating the standardized coefficient weights and 95% CIs for the second step of the hierarchical regression analyses predicting the recognition memory task outcome variables. H − FA represents the difference between rates of hits and false alarms, an index of memory accuracy

We explored the relationship between motivation and recognition memory, using the RMT (Cortese et al., 2010; 2015). There were 2,595 words with values for all variables of interest. For hits, all control variables, except AoA, were significant predictors, with more hits for words that were shorter, less frequent, and more orthographically unique. The addition of the semantic variables significantly improved the model fit, explaining an additional 11.2% of variance. Only concreteness, valence, and semantic diversity were significant predictors, with participants tending to correctly recognize words that were more concrete, more extremely valenced, and less semantically diverse. When predicting the number of false alarms, all control variables, except for frequency, were significant predictors, with higher false alarm rates for longer, more orthographically similar, and later acquired words. The addition of the semantic variables significantly improved the model fit, explaining an additional 8.3% of variance. Here, all variables were significant predictors of the rate of false alarms. Words tended to be falsely recognized if they were less concrete, less extremely valenced, more semantically diverse, and more strongly associated with motivation. Finally, all control variables were significant predictors of the difference between hits and false alarms (indexing memory accuracy), with less accurate memory for words that were longer, more frequent, more orthographically similar, and later acquired. The addition of the semantic variables significantly improved the model fit, explaining an additional 20.7% of variance, where all variables were significant predictors. Here, memory was less accurate for words that were less concrete, less extremely valenced, more semantically diverse, and strongly associated with motivation (see Fig. 5 for illustrated standardized coefficients).

### Composite measure

Finally, we computed a composite measure of motivation and emotional valence (Warriner et al., 2013) to provide an estimate of a more general reward construct, and repeated the above hierarchical regression analyses with the composite instead of the separate variables to explore whether it better accounted for variance in behavioural data.

Following the methodology from Lynott et al. (2019), we used four methods to reduce the motivation and valence variables into a single composite variable that could be used in further studies as a measure of reward. We used Minkowski (*m* = 3) distance, Euclidean distance, the sum of all ratings, and principal component analysis (PCA) component scores. We included each of these composite variables, along with concreteness and semantic diversity, in eight separate regression models predicting LDT, ECP, SDT, and RMT outcomes and compared the models’ performance against a null model (the first step from the previous analyses containing lexical control variables), while excluding motivation and valence. While all models significantly improved the model fit and explained additional variance to that explained by the control variables, neither of the candidate composite variables accounted for additional variance to that previously explained by the two individual semantic variables (motivation and valence). See Section S7 of the supplementary materials for regression tables.

## Discussion

Contemporary research into the systems underpinning semantic representation is increasingly moving beyond the established contributions of sensory and motor experience and turning its attention more towards endogenous sources of information, including cognitive and affective processes (Barsalou, 1999); Binder et al., 2016). Reward processing (inclusive of affective and motivation components; Berridge & Robinson, 2016) is a highly salient feature of many human experiences that likely shapes the way we attribute meaning. Yet, its contribution to semantic cognition has seldom been explored. With the aim of laying the groundwork for future endeavours, we collected normative ratings of associations with reward for a large set of words. This resulted in a novel set of norms that capture associations with *motivation* specifically, and for 8,601 words, including nouns, verbs, and adjectives that also had pre-existing emotion ratings (Warriner et al., 2013). The motivation ratings show high reliability, which suggests they capture a meaningful construct across participants. Validity was also confirmed by a strong correlation of the new motivation ratings with those on a similar ‘drive’ attribute measured by Binder et al. (2016), despite some differences in the definitions employed. Further, we performed analyses that showed that the motivation dimension (a) captures a distinct aspect of word meaning when compared alongside other established semantic variables, as well as lexical variables, and (b) can account for variance in behavioural responses in lexical-semantic tasks. Finally, we explored the utility of a composite variable, derived from a combination of motivation and emotional valence ratings, aimed at approximating a more general ‘reward’ construct.

Correlations between the new motivation ratings and various other semantic properties of the words provided initial insights into the nature of the motivation dimension. The motivation measure was most strongly correlated with Binder et al.’s (2016) ‘drive’ attribute, which was proposed to ‘represent the degree of general motivation associated with a target concept’ (p. 142), so this is both unsurprising and validating. However, these two measures shared 53% of their variance, which suggests our definition could be capturing a greater variety of motives or drive experiences, as was intended. For example, it is possible that rating associations of a word with ‘a sense of desire to achieve or obtain a goal’ captures a greater sense of agency as well as higher-order cognition (i.e., goal-directed or model-based behaviour) than asking whether a word is ‘something or someone that makes you do something’ (as per Binder et al., 2016) which might be taken to imply more habitual forms of behaviour (Drummond & Niv, 2020). This possibility would need to be explored in future research that uses a property-generation task or a similar approach (e.g., Wiemer-Hastings & Xu, 2005). The correlation coefficient revealed a much weaker association between the motivation measure and Binder and colleagues’ other drive-related ‘needs’ attribute. The latter was measured by asking whether a word refers to ‘something or someone you could not live without’ and thus refers specifically to the goal of a behaviour, rather than the drive state associated with fulfilling it. While it is conceptually difficult to fully disentangle motivation/drive from the goal to which it is directed, these two measures do at least appear to be capturing different aspects of word meaning. Future research will be needed to further explore whether they could relate to different elements of reward experience. Alternatively, the distinction could reflect the way in which people relate certain aspects of reward experience to different types of concepts (e.g., more embodied concepts such as *food*, versus less-embodied concepts, like *success*; also see below).

We found that words with strong associations to motivation tend to be more valenced, arousing, and dominant. These correlations mirror the close relationship between drive experiences and affect that features at the heart of debate in the reward literature (Havermans, 2011; Pool et al., 2016). Emotions are often elicited when a need/goal, and the drive to obtain it, is either satisfied or thwarted. They are often a dominant aspect of reward experience, but they are not the brain’s only response, as it cascades into higher-order cognitive processing and learning (Berridge et al., 2009). Importantly, the correlations we observed between the motivation ratings and emotion-related measures are modest, and this suggests that the former cannot be completely accounted for by the latter when it comes to capturing word meaning. Indeed, this finding supports the claim that drive experience contributes to semantic representation in a way that is distinct from affective experience. It may be, therefore, that affect ratings and motivation ratings tap into different mechanisms that underpin reward processing (e.g., liking versus wanting), but this will need to be explored by further research that focuses on the types of features associated with words with high versus low motivation ratings.

Words with high motivation ratings also tend to have low concreteness, low imageability, low sensory–experience and low body–object interaction ratings, suggesting they rely less on sensorimotor information. These correlations are moderate, however, so the motivation measure is not merely capturing abstractness of concepts per se. Multiple representation theories suggest concepts that are more abstract and, by definition, are not tangible or observable, rely on other sources of semantic information, including experience of affect, language, and social interaction (e.g., Barsalou, 2020). Similarly, reward/drive experiences could be involved in representing all manner of concepts but, at the same time, be particularly important for abstract concepts (see below for further discussion). Words with high motivation ratings also tend to be more semantically diverse. Semantic diversity is a measure of semantic ambiguity based on how words are used in different linguistic contexts (Hoffman et al., 2013). It is readily apparent how the highest-rated words on motivation (see Table 2) could apply to a diverse number of contexts, and how they are also less categorically specific (see Bolognesi et al., 2020) compared to the lowest-rated words (e.g., *career* versus *bellman*). Not surprisingly, conceptual specificity is positively correlated with concreteness (Villani et al., 2024), and semantic diversity is negatively correlated with concreteness (Hoffman et al., 2013). Thus, the relationship between motivation ratings and semantic diversity may reflect the fact that the highest rated words on motivation tend to be quite abstract. Importantly, and like that with concreteness, the correlation of the motivation ratings with semantic diversity is only modest. Therefore, it does not completely account for the motivation measure, which is, to an extent, capturing distinct aspects of word meaning.

It is noteworthy that the overall pattern of findings surrounding the new motivation measure (including the ability to explain variance in behavioural responses; the facilitated decisions, but poorer recall discussed below) is similar to that found by Muraki et al. (2022) for a set of verbs that describe mental states. Words highly rated for motivation not only tended to be verbs rather than nouns but were also often words that pertain to mentality (e.g., *achieve, aspire*, *improve*). Nouns such as ‘income’ were rated highly on motivation, whereas other arguably reward-laden nouns like ‘chocolate’ were rated lower. This is also taken to suggest that the motivation measure is capturing more broad-scope goal-related associations, as opposed to object-focused pleasure-related associations, and these include mental outlook/attitudes. However, our study provides only initial insights into the information underlying motivation ratings, and subsequent work will be needed to identify the specific aspects of drive experiences that relate to conceptual processing.

We found that the degree to which a word’s meaning is associated with motivation is related to performance in lexical tasks. Importantly, this was true after controlling for other semantic variables known to influence lexical-semantic processing, namely concreteness, valence extremity, and semantic diversity. Moreover, facilitatory effects of the motivation ratings (faster decision latencies and better accuracy) were observed both in tasks that require explicit semantic judgements (abstract responses in the SDT) and in tasks that involve only shallow semantic processing, such as the word knowledge task and LDT. In these latter tasks, there is limited variance left after the inclusion of lexical variables (e.g., frequency and length) to be explained by semantic predictors. The unique and pervasive contribution made by the motivation measure suggests it captures important information about the semantic representation of words and appears consistent with prior research on semantic richness effects (Pexman, 2012), and the idea that ‘more is better’ (Balota et al., 1991, p. 214). Semantic richness refers to a phenomenon by which processing for words that are associated with relatively more semantic information is facilitated in lexical and semantic tasks (Pexman, 2012). This is thought to reflect richer conceptual representations that result in more semantic activation and/or faster semantic settling, and can provide stronger feedback into orthographic processes, thereby allowing faster and more accurate lexical decisions (Hino et al., 2002; Hino & Lupker, 1996). Likewise, semantically rich words might elicit more elaborative encoding, which is thought to improve recognition (Lockhart & Craik, 1990). The motivation measure is one of several semantic variables that can predict behavioural performance, and thus our results are also consistent with theories that claim that multiple kinds of information simultaneously contribute to the richness of conceptual representations and facilitate lexical-semantic processing, including linguistic (i.e., semantic diversity), sensorimotor (i.e., concreteness), and affective experience (i.e., valence; Barsalou et al., 2008; Borghi et al., 2019). We provide the first evidence to date that motivation/drive experience is one of these sources of information.

Nonetheless, the effect of motivation ratings on behavioural performance was not ubiquitous in all tasks. First, the facilitatory effect of rated motivation in the SDT was specific to semantic decisions about abstract words and was not observed for decisions about concrete words. This aligns with previous research that revealed how the influence of some other semantic dimensions on lexical-semantic performance is dependent on word concreteness (Diveica et al., 2024a, b; Newcombe et al., 2012). The processing of concrete words is mainly facilitated by concreteness (ergo, sensorimotor experience). However, abstract word processing benefits from higher emotional valence and greater semantic diversity (Newcombe et al., 2012; Pexman & Yap, 2018; Pexman et al., 2017). Indeed, some multidimensional theories argue that non-sensory experience-based semantic dimensions (e.g., emotional and social information) are particularly key to the acquisition and grounding of abstract concepts (e.g., Barsalou, 2020; Borghi et al., 2019). In the same manner, drive experience may be especially important for enriching the representation of concepts with less direct association with sensory features. However, further research is needed to better understand the componential manner in which these different forms of experience contribute to the semantic richness of concepts (for examples of studies examining the factor structure of semantic richness, see Diveica et al., 2024a; Muraki et al., 2020). Second, words with high motivation ratings tended to be associated with poorer memory accuracy and higher rates of false alarms in the recognition memory task. This may also reflect the fact that words with low motivation ratings tend to be less concrete. However, in this case, the effect of motivation is probably less to do with enrichment of a word’s conceptual representation, and more to do with the similarity in meaning amongst concepts that are highly associated with motivation which impacts the ability to distinguish between old and new words (Muraki et al., 2022). Overall, these interpretations are consistent with a dynamic view of semantic processing according to which the effects of semantic variables on lexical processing are task-dependent and sometimes reflect the fact that participants tend to focus on dimensions of meaning that are relevant to the specific decision (Newcombe et al., 2012; Tousignant & Pexman, 2012). Future studies should consider the dimension of motivation to further our understanding of abstract concept representation.

Finally, we applied several methods towards computing a composite ‘reward’ variable, derived from a combination of motivation and emotional valence ratings (cf. Berridge & Robinson, 2016). We found that regression models including these composites as predictors explain unique variance in behavioural performance on lexical and semantic tasks, and this suggests that a composite could be useful in analytic contexts where multicollinearity needs to be minimized, for example. In such cases, we recommend the use of that computed via the Minkowski distance method, as this composite consistently outperformed the other three candidate variables. However, these models did not outperform those utilising the separate ratings of motivation and emotional valence. The two measures do capture overlapping processes (as is shown via correlations), and thus they could be interacting in a way that reflects a latent ‘reward’ construct. Future research is needed to test this directly with methods such as structural equation modelling. Indeed, Binder et al.’s (2016) exploratory factor analysis of several experiential attributes extracted a latent variable for which the highest loading variables approximated reward value (benefit, needs) and motivation (drive). There are several methodological differences between the present study and that of Binder et al (2016) that warrant consideration. First, Binder et al. analysed only 496 words, containing nouns and verbs (*N* = 62), whereas our analysis contained 6,860 words and several common parts of speech. Their dataset was therefore more vulnerable to spurious correlations and/or effects specific to a limited subset of concepts. Second, ratings in our study were obtained only on a single measure, whereas Binder et al.’s participants rated each word on 65 different attributes. Under these latter conditions, ratings on one attribute are more likely to influence ratings on another. Finally, and perhaps most importantly, the definitions used by Binder and colleagues have different emphases from our motivation definition and those used in Warriner et al.’s (2013) affect ratings study. Therefore, they might be capturing different aspects of the reward experience. This highlights the need for future research that teases apart the way reward/drive states are experienced for different types of concepts and the need to refine these definitions/constructs.

## Conclusion

In the present study, we compiled a new, openly available set of ratings that capture the association of motivation/drive experiences with a word’s meaning. We used a definition of motivation that was intended to capture a variety of motives and reward experiences, ranging from the emotional to the social and the intellectual. It produced reliable ratings, and those ratings were a significant predictor of performance in several behavioural tasks (having controlled for other lexical and semantic variables), demonstrating that the measure is related to conceptual representations. Our study also provides some initial insights into the semantic information captured by the motivation measure, and this appears to be related to goal-related associations, but subsequent work will be needed on this matter. Indeed, the motivation norms will enable future research into the organisation and grounding of conceptual knowledge and can help target testable predictions about brain and behaviour. This includes the role and behavioural consequences of different types of meaning-imbued experience across the lifespan, including during acquisition, retrieval and when the semantic system is impaired. For example, research in the neuropsychological domain has shed light upon the relationship between reward-related systems and the semantic system in that disturbances to stimulus-reward associations appear to contribute to profound social-semantic impairments characteristic of frontotemporal dementia (for a related review, see Rankin, 2020; Rouse et al., 2024). Using these norms along with functional neuroimaging, it may be possible to glean a better understanding of how brain regions implicated in decision making and reward processing (e.g., the orbitofrontal and ventromedial frontal cortex; Lockwood et al., 2024; Rudebeck & Rich, 2018) interact with the anterior temporal semantic hub (Binney & Ramsey, 2020; Lambon Ralph et al., 2017) in a way that is not possible with patient data.

## Supplementary Information

Below is the link to the electronic supplementary material.

Supplementary file1 (DOCX 1.05 MB)

## Data Availability

The datasets generated during and/or analysed during the current study are available in the OSF repository (https://osf.io/3vx9y/).
