# Supplementary material for: Reward as a facet of word meaning: Ratings of motivation for 8,601 English words
Source: Behav Res Methods. 2025 Jul 29;57(9):242. doi: 10.3758/s13428-025-02762-8 (PMC12307516; doi:10.3758/s13428-025-02762-8)
Supplement: Supplementary file 1 — Supplementary file1 (DOCX 1.05 MB) [file 13428_2025_2762_MOESM1_ESM.docx]

**Supplementary Materials**

for

**Reward As a Facet of Word Meaning: Ratings of Motivation for 8601 English Words**

Doina-Irina Giurgea, Penny M. Pexman, Richard J. Binney

Contents:

**Section S1 Preliminary Analyses of a Reward Factor identified by Binder et al., (2016) (p. 2)**

**Section S2: Pilot Word Ratings Study (p. 6)**

**Section S3: Pilot Stimuli (p. 15)**

**Section S4: Motivation Rating Instructions (p. 17)**

**Section S5: Additional Results for Hierarchical Regressions (p. 19)**

**Section S6: Scatterplots of the Correlation of Motivation with other Psycholinguistic Dimensions (p. 30)**

**Section S7: Evaluation of the Composite Measure (p. 31)**

**Section S8: Arousal and Dominance (p. 43)**

**Section S1. Preliminary Analyses of a Reward Factor identified by Binder et al. (2016)**

All analyses were implemented in RStudio (version 4.3.3; 2024; psych package version 2.4.6.26). In an initial step, we attempted to replicate the results of the factor analysis performed by Binder et al. (2016) by closely following the methodology outlined in their paper. We extracted 16 factors, with loadings restricted to >.31. Of these factors, one emerged with the dimensions benefit, needs, and drive loading onto it, which was interpreted as a Reward factor. For each word item we extracted the reward factor score to include in subsequent regression analyses.

To explore the relationships between behavioural performance on lexical tasks and the reward factor identified by Binder et al. (2016), we performed a series of hierarchical regression analyses. The behavioural data were from the lexical decision task (LDT) in the English Lexicon Project (ELP; Balota et al., 2007; reaction time and error rates when required to indicate whether a stimulus is a word or a non-word) and the English Crowdsourcing Project (ECP) word knowledge task (Mandera et al., 2020; reaction time and word prevalence when participants are required to indicate whether they know a word presented to them). There were 396 items for which we had data for all variables of interest. We entered predictor variables in our model in two steps: 1) control variables, including word length, frequency (log transformed SUBTLEX; Brysbaert & New, 2009), and AoA (Kuperman et al., 2012); 2) semantic variables, including concreteness (Brysbaert et al., 2014), valence extremity (Warriner et al., 2013), semantic diversity (Hoffman et al., 2013), and the reward factor. In a final step, we substituted the reward factor for the individual reward-related variables (benefit, needs, drive), to assess their individual ability to explain variance. All predictors were mean-centred.

In the first step of analysing LDT performance, we entered the control variables and observed that they were all significant predictors of LDT reaction times, with faster reaction times (RTs) being associated with words that are shorter, more frequent, and acquired earlier. The addition of the semantic variables did not improve the model fit. When predicting LDT error rates, all control variables were significant predictors, with fewer errors for words that were longer, more frequent, and acquired earlier. The addition of the semantic variables did not significantly improve the model fit. When replacing the reward factor with the individual reward-related variables, we did not identify an improvement in model fit (see Table S1 for a summary of the models).

**Table S1.** *Regression coefficients from item-level analyses predicting lexical decision task reaction times and error rates (N=396)*

| zRT | | | | | | | Error rates | | | | | | | |
| --- | --- | --- | --- | --- | --- | --- | --- | --- | --- | --- | --- | --- | --- | --- |
| Predictor | b | SE | t | p | sr2 | R2 | ∆R2 | b | SE | t | p | sr2 | R2 | ∆R2 |
| Step1 |  |  |  |  |  | 0.59 | 0.59 |  |  |  |  |  | 0.15 | 0.15 |
| Intercept | -0.517 | 0.008 | -67.39 | *** |  |  |  | 0.033 | 0.002 | 14.61 | *** |  |  |  |
| Length | 0.041 | 0.004 | 9.65 | *** | 0.096 |  |  | -0.004 | 0.001 | -3.36 | ** | 0.024 |  |  |
| Frequency | -0.148 | 0.013 | -11.4 | *** | 0.135 |  |  | -0.018 | 0.004 | -4.75 | *** | 0.049 |  |  |
| Age of Acquisition | 0.019 | 0.004 | 4.99 | *** | 0.026 |  |  | 0.004 | 0.001 | 3.72 | *** | 0.03 |  |  |
| Step2 |  |  |  |  |  | 0.59 | -0.001 |  |  |  |  |  | 0.16 | 0.011 |
| Intercept | -0.517 | 0.008 | -67.3 | *** |  |  |  | 0.033 | 0.002 | 14.71 | *** |  |  |  |
| Length | 0.042 | 0.004 | 9.66 | *** | 0.097 |  |  | -0.004 | 0.001 | -2.88 | ** | 0.018 |  |  |
| Frequency | -0.138 | 0.016 | -8.9 | *** | 0.082 |  |  | -0.013 | 0.005 | -2.94 | ** | 0.018 |  |  |
| Age of Acquisition | 0.02 | 0.005 | 4.12 | *** | 0.018 |  |  | 0.004 | 0.001 | 3.04 | ** | 0.02 |  |  |
| Concreteness | 0 | 0.01 | -0.04 | 0.967 | 0 |  |  | -0.002 | 0.003 | -0.73 | 0.464 | 0.001 |  |  |
| Valence Extremity | -0.006 | 0.011 | -0.51 | 0.608 | 0 |  |  | -0.002 | 0.003 | -0.65 | 0.517 | 0.001 |  |  |
| Semantic Diversity | -0.016 | 0.039 | -0.41 | 0.684 | 0 |  |  | -0.012 | 0.012 | -1.05 | 0.295 | 0.002 |  |  |
| Reward | -0.014 | 0.008 | -1.67 | 0.096 | 0.003 |  |  | -0.007 | 0.002 | -2.83 | ** | 0.017 |  |  |

*Note*. b represents the unstandardized beta coefficients. SE represents the standard error of the beta coefficients. sr^2^ represents the semi-partial correlation squared. zRTs refers to the standardized reaction times. **p*<.05; ***p*<.01; ****p*<.001

We next analysed ECP word knowledge task performance. When predicting RTs, all control variables were significant predictors, with faster reaction times for shorter, more frequent, and early acquired words. The addition of the semantic variables (including the reward factor) significantly improved the model fit, explaining an additional 1% of variance. Here, only the reward factor and valence extremity were significant predictors, with shorter reaction times for words more extremely valenced and with higher scores on the reward factor. When predicting the proportion of participants reporting not knowing a word, all control variables were significant predictors, with less prevalent words being shorter, less frequent, and acquired later. The addition of the semantic variables did not significantly improve the model fit. When replacing the reward factor with the individual reward-related variables, we did not identify an improvement in model fit (see Table S2 for a summary of the models).

**Table S2.** *Regression coefficients from item-level analyses predicting ECP word knowledge task reaction times and proportion of people reporting not knowing a word (N = 396)*

| zRT | | | | | | |  | Proportion unknown | | | | | | |
| --- | --- | --- | --- | --- | --- | --- | --- | --- | --- | --- | --- | --- | --- | --- |
| Predictor | b | SE | t | p | sr2 | R2 | ∆R2 | b | SE | t | p | sr2 | R2 | ∆R2 |
| Step1 |  |  |  |  |  | 0.41 | 0.41 |  |  |  |  |  | 0.2 | 0.2 |
| Intercept | -0.626 | 0.003 | -222.24 | *** |  |  |  | 0.007 | 0.001 | 13.07 | *** |  |  |  |
| Length | 0.006 | 0.002 | 4.15 | *** | 0.025 |  |  | -0.001 | 0 | -3.79 | *** | 0.029 |  |  |
| Frequency | -0.044 | 0.005 | -9.13 | *** | 0.124 |  |  | -0.006 | 0.001 | -6.65 | *** | 0.09 |  |  |
| Age of Acquisition | 0.006 | 0.001 | 4.18 | *** | 0.026 |  |  | 0.001 | 0 | 3.21 | ** | 0.021 |  |  |
| Step2 |  |  |  |  |  | 0.42 | 0.01 |  |  |  |  |  | 0.21 | 0.008 |
| Intercept | -0.626 | 0.003 | -224.08 | *** |  |  |  | 0.007 | 0.001 | 13.14 | *** |  |  |  |
| Length | 0.007 | 0.002 | 4.52 | *** | 0.03 |  |  | -0.001 | 0 | -3.48 | ** | 0.024 |  |  |
| Frequency | -0.04 | 0.006 | -6.99 | *** | 0.071 |  |  | -0.005 | 0.001 | -4.85 | *** | 0.047 |  |  |
| Age of Acquisition | 0.007 | 0.002 | 3.81 | *** | 0.021 |  |  | 0.001 | 0 | 3.44 | ** | 0.024 |  |  |
| Concreteness | 0.002 | 0.004 | 0.59 | 0.553 | 0.001 |  |  | 0.001 | 0.001 | 0.95 | 0.341 | 0.002 |  |  |
| Valence Extremity | -0.009 | 0.004 | -2.37 | ** | 0.008 |  |  | 0 | 0.001 | -0.15 | 0.879 | 0 |  |  |
| Semantic Diversity | 0.007 | 0.014 | 0.49 | 0.624 | 0 |  |  | 0.001 | 0.003 | 0.26 | 0.795 | *** |  |  |
| Reward | -0.007 | 0.003 | -2.22 | * | 0.007 |  |  | -0.001 | 0.001 | -2.62 | 0.009 | ** |  |  |

*Note*. b represents the unstandardized beta coefficients. SE represents the standard error of the beta coefficients. sr^2^ represents the semi-partial correlation squared. zRTs refers to the standardized reaction times. **p*<.05; ***p*<.01; ****p*<.001

**Section** **S2. Pilot Word Rating Study**

**Motivation**

***Participants***

We piloted the motivation rating task with 20 participants (12 male, 7 female, 1 other, M_age_ = 38.5, SD_age_ = 14.85) recruited via Prolific. No data were excluded. English was the first language for all participants, with 18 (90%) of them reporting being monolingual, and 2 (10%) reporting speaking more than one language. Participants completed the rating task in 21 minutes on average and were compensated with GBP £4.50.

***Materials***

We selected 60 words spanning the dimensions of concreteness (Brysbaert et al., 2014), valence (Warriner et al., 2013), and drive (Binder et al., 2016; see Table S3 in Section S3). We defined motivation as a sense of desire to achieve or obtain a goal (see Section S4 for full instructions).

***Procedure***

The word stimuli were presented using Qualtrics and linked to the Prolific recruitment platform. Following the consent form, demographics survey, and instructions, the participants rated how well they understood the instructions on a 5-point scale ranging from “extremely well” (1) to “not well at all” (5). They were also asked to indicate whether they found any aspect of the instructions unclear and confusing, and, if so, what aspect. Then, the participants rated all the items using a 7-point Likert scale presented horizontally below each word. In addition, there was an “I don’t know the meaning of this word” option. Items were presented in random order and only one word was presented per page. For 30% of the items, participants were prompted to explain the reasoning behind their chosen rating by typing their answer in a text box. Finally, at the end of the ratings task, participants were asked to explain the task instructions using a text box.

***Results***

*Understanding of Instructions*

The data and analysis scripts can be accessed via the OSF project page (https://osf.io/3vx9y/). Ten percent of the pilot participants reported understanding the instructions moderately well, 50% very well and 40% extremely well. Most participants provided explanations that were consistent with our definition (e.g., “for a word to relate to motivation it has to be something that can be associated with it like words such as guilt and regret as they may be a factor for motivation”; “something which drives you to do something else or the reason behind something occurring”; “for a word to relate to 'motivation' it must be a contributory factor in the level of motivation a person experiences whether that be to make a person more or less inclined to take action”). Some participants clearly identified a distinction between our measure and emotional valence, indicating that the motivation rating was independent of the valence associated with the word (e.g., “does not matter if it is positive or negative”).

*Reliability*

We computed the reliability of ratings using split half reliability for the 60 words. We found a mean Spearman-Brown corrected split-half reliability of 0.94 (SD = 0.15) across 100 random splits, which indicated excellent reliability. We computed the intraclass correlation coefficient using a two-way (random effects) model based on agreement. We examined the reliability of ratings from a typical, single rater and we found an ICC (2,1) = 0.36, 95% CI [0.28;0.47], indicating poor reliability. We also examined the reliability of ratings from the 20 raters averaged together and we found an ICC(2,20) = 92, 95 % CI [0.89;0.95], indicating excellent reliability.

*Relation to existing word norms*

We explored the relationship between our pilot ratings of motivation and Binder et al.’s (2016) drive ratings. We found a moderate positive correlation (*r*= 0.58, R^2^ = 0.33, *p*= 0.005; 33% variance shared). Moreover, there was a positive correlation between the motivation ratings and emotional valence ratings (Warriner et al., 2013; *r*= 0.56, R^2^ = 0.31, p <.001; 31% variance shared). The motivation ratings were not significantly correlated with positive valence (words with a rating above 5 in the Warriner et al. (2013) affect ratings data set; *r*=0.27, R^2^ =0.07, *p*=0.097; 7% variance shared). Overall, these observations suggested that our measure of motivation captured information that was partially distinct from these previously described norms.

**Pleasure and Reward**

***Participants***

A total of 40 participants (14 male, 26 female, M_age_ = 36.1, SD_age_ = 11.83) completed the study. There were two versions of the participant instructions, and 20 participants saw version 1 (referring to the dimension of interest as ‘pleasure’; see below) and 20 saw version 2 (referring to the dimension of interest as ‘reward’). No data were excluded. English was the first language for all participants, with 25 (62.5%) reporting being monolingual, and 15 (37.5%) reporting speaking more than one language. Participants completed the rating task in 22 minutes on average and were compensated with GBP £4.50.

***Materials***

We selected 60 words spanning the dimensions of concreteness (Brysbaert et al., 2014) and valence (Warriner et al., 2013; see Table S4 in Section S3 for stimuli). We created two versions of the instructions that differed in their use of the words “pleasure” or “reward”, and the example words provided that should have high, low, and intermediate ratings (see Table S4 in Section S3). Otherwise, the instructions were identical. This was done with the aim of identifying terminology that would prompt evaluation of cognitive experiences of reward in addition to experiences of emotion. In Version 1, we defined pleasure as “the experience of a sense of *enjoyment* or satisfaction in association with something, which is broadly beneficial”. In Version 2, we defined reward as “the experience of a sense of *pleasure* or satisfaction in association with something, which is broadly beneficial.”

***Procedure***

We used the same procedure as in the pilot of motivation ratings.

***Results***

*Understanding of Instructions*

The data and analysis scripts can be accessed via the OSF project page (https://osf.io/3vx9y/). Twenty percent of participants reported understanding the instructions very well and 80% extremely well for the pleasure version of the instructions. For the reward version, 40% of participants reported understanding the instructions very well, while 60% reported understanding the instructions extremely well. Most participants provided explanations that were consistent with our definition (e.g., “how it relates to physical pleasure, how it relates to something emotionally, like happiness is a pleasurable feeling, or the things that the word can be associated with. For example, holiday would be associated with happiness and relaxation which are pleasurable things”; “something that is enjoyable, beneficial, or life enhancing in some way”; “Something that gives you satisfaction on a mental level, on a physical level or by some sort of act that you undertake or even receive from others.”). The participants often associated pleasure with reward, even if reward was not mentioned in the instructions for the “pleasure” version of the instructions (“A word may be considered pleasurable if it brings some reward”). In addition, many participants directly expressed an association with emotional valence (e.g., “Pleasure means that the word is associated with something positive, whether it actually is something pleasurable and positive or implies something positive”). The results were similar for the reward version of the instructions, where participants provided explanations consistent with our definitions (e.g., “Reward is usually associated with many positive experiences and in most instances leads to some sort of benefit. Each experience can be different, it could be mental, emotional or a physical reward.”), and showed a direct link to positive experiences (e.g., “For a word to relate to reward it needs to have positive connotations and be associated with some kind of achievement, joy, or pleasure”).

*Instructions Version*

We conducted a Pearson’s Chi-Square test of independence to assess the self-reported understanding of the two versions of the instructions. There was no significant difference between the two versions in the self-reported understanding scores of the instructions, χ^2^(1) = 1.071, *p* = .301. There was a strong positive correlation between the means of ratings of the two different instruction conditions, *r*= 0.96, R^2^= 0.92, *p* <.001). We conducted a two-sample t-test to explore the effect of version on the mean ratings of the words. This revealed no significant effect of instruction version on the mean ratings (*t*(117.41) = -0.22, *p*= 0.83, CI[-0.72; 0.57]. We also found no significant effect of instruction version on the standard deviation of the ratings (*t*(117.12) = -0.99, *p*= 0.33, CI[-0.27; 0.09]). These results suggest that the terminology used did not significantly influence the way participants rated the stimuli.

We also explored the relationship between our ratings of pleasure/reward and available emotion ratings (Warriner et al., 2013). The correlation between the pleasure ratings and emotion valence was significant and positive (*r*= 0.94, R^2^ = 0.87, *p* <.001; 87%), as was the relationship between the reward ratings and emotion valence (*r*= 0.9, R^2^ = 0.81, *p* <.001; 81%). We also explored this relationship using only positively valenced words (i.e., only words with a rating above 5 in the Warriner et al. (2013) affect ratings data set). Here, both pleasure (*r*= 0.77, R^2^ = 0.59, *p* <.001; 59%) and reward (*r*= 0.66, R^2^ = 0.43, *p* <.001; 43%) had a strong positive correlations with positive emotional valence. These results suggested that our measures and emotional valence captured much of the same information.

*Reliability*

Given the strong correlation between ratings resulting from Version 1 and Version 2 of the instructions we combined the data to compute reliability of the ratings. This was done using split half reliability for the 60 words. We found a mean Spearman-Brown corrected split-half reliability of 0.95 (SD = 0.12) across 100 random splits, which indicated excellent reliability. In addition, we assessed inter-rater reliability by computing the two-way random-effects intra-class correlation coefficient (ICC) based on absolute agreement. We examined the reliability based on the ratings from one typical, single rater and we found an ICC(2,1) =0.58, 95% CI [0.49, 0.67] suggesting moderate reliability. We also examined the reliability for the average ratings of 40 raters and we found an ICC(2,40) = .98, 95% CI [0.98, 0.99], indicating excellent reliability. When examining the ratings for the version used in the main experiment (Version 1), we found an ICC of 0.94, 95% CI [0.91, 0.96]) which suggested that the average ratings of 20 raters were highly reliable.

**Instructions**

***Pleasure Ratings***

We frequently encounter pleasure in many forms, during our daily lives. This study is concerned with objects, events, and interactions (and qualities of these things) that we experience and perceive as being pleasurable.

The purpose of this study is to rate the extent to which words, and their referents, are associated with pleasure. There are many ways in which a word could refer to something ‘pleasurable’. To experience ‘pleasure’ is to experience a sense of enjoyment or satisfaction in association with something. More broadly though, to be ‘pleasurable’, is to be beneficial in some sense.

Words can refer to a token of pleasure (e.g., “money”, “trophy”). They can refer to something that is ‘pleasurable’ in a physical sense (e.g., “food”, “sex”), or an emotional sense (e.g., “happiness”, “love”), but pleasure can also be experienced in a cognitive or intellectual way (e.g., to “achieve”, to “discover” or to “solve”). In addition, pleasure can be experienced by interacting with others (e.g., “cooperation”, “conversation”, “support”), as well as individually (e.g., “promotion”, “pride”). While some things and experiences are almost always pleasurable (e.g., “sugar”, “winning”), some are more situation-dependent (e.g., to “read”, “competition”). Some pleasurable experiences are immediate (e.g., to “eat”) but others can be delayed, or perhaps even uncertain (e.g., “investment"). In contrast, there are things that are not at all pleasurable (e.g., “hurricane”). We are interested in exploring the extent to which words of all kinds are perceived as being related to pleasure.

In this study, you are required to rate words based on the degree to which they have a meaning that is associated with ‘pleasure’. Any word (e.g., “ice cream”) that in your estimation refers to something you perceive as being highly associated with pleasure should be given a high pleasure rating (at the upper end of the numerical scale). Any word (e.g., “brick”) that in your estimation refers to something that lacks an association with pleasure should be given a low pleasure rating (at the lower end of the scale). Any word that in your estimation refers to something that is not fundamentally pleasurable but has some pleasurable elements (e.g., ‘driving’), or can be thought of as pleasurable in some circumstances (e.g., ‘lecture’), should be given an intermediate pleasurable rating.

Please make your ‘pleasure’ ratings using the 7-point scale. A value of 1 indicates a low ‘pleasure’ rating, and a value of 7 indicates a high ‘pleasure’ rating. The values ranging from 2 to 6 are intermediate ratings. Please feel free to use the whole range of values provided when making your ratings. Click on the rating that is most appropriate for each word. When making your ratings, try to be as accurate as possible, but do not spend too much time on any one word. If you are not familiar with a word’s meaning, please select ‘I don’t know the meaning of this word’.


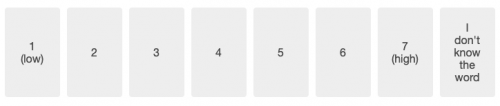


***Reward Ratings***

We frequently encounter rewards in many forms, during our daily lives. This study is concerned with objects, events, and interactions (and qualities of these things) that we experience and perceive as being rewarding.

The purpose of this study is to rate the extent to which words, and their referents, are associated with reward. There are many ways in which a word could refer to something ‘rewarding’. To experience ‘reward’ is to experience a sense of pleasure or satisfaction in association with something. More broadly though, to be ‘rewarding’, is to be beneficial in some sense.

Words can refer to a token of reward (e.g., “money”, “trophy”). They can refer to something that is ‘rewarding’ in a physical sense (e.g., “food”, “sex”), or an emotional sense (e.g., “happiness”, “love”), but reward can also be experienced in a cognitive or intellectual way (e.g., to “achieve”, to “discover” or to “solve”). In addition, reward can be experienced with others (e.g., “cooperation”) or through others (e.g., “conversation”, “support”), as well as individually (e.g., “promotion”, “pride”). While some things and experiences are almost always rewarding (e.g., “sugar”, “winning”), some are more situation-dependent (e.g., to “read”, “competition”). Some rewards are experienced immediately (e.g., to “eat”) but others can be delayed, or perhaps even uncertain (e.g., “investment"). In contrast, there are things that are not at all rewarding (e.g., “hurricane”). We are interested in exploring the extent to which words of all kinds are perceived as being related to reward.

In this study, you are required to rate words based on the degree to which they have a meaning that is associated with ‘reward’. Any word (e.g., “ice cream”) that in your estimation refers to something you perceive as being highly associated with reward should be given a high reward rating (at the upper end of the numerical scale). Any word (e.g., “brick”) that in your estimation refers to something that lacks an association with reward should be given a low reward rating (at the lower end of the scale). Any word that in your estimation refers to something that is not fundamentally rewarding but has some rewarding elements (e.g., ‘driving’), or can be thought of as rewarding in some circumstances (e.g., ‘lecture’), should be given an intermediate reward rating.

Please make your ‘reward’ ratings using the 7-point scale. A value of 1 indicates a low ‘reward’ rating, and a value of 7 indicates a high ‘reward’ rating. The values ranging from 2 to 6 are intermediate ratings. Please feel free to use the whole range of values provided when making your ratings. Click on the rating that is most appropriate for each word. When making your ratings, try to be as accurate as possible, but do not spend too much time on any one word. If you are not familiar with a word’s meaning, please select ‘I don’t know the meaning of this word’.

**
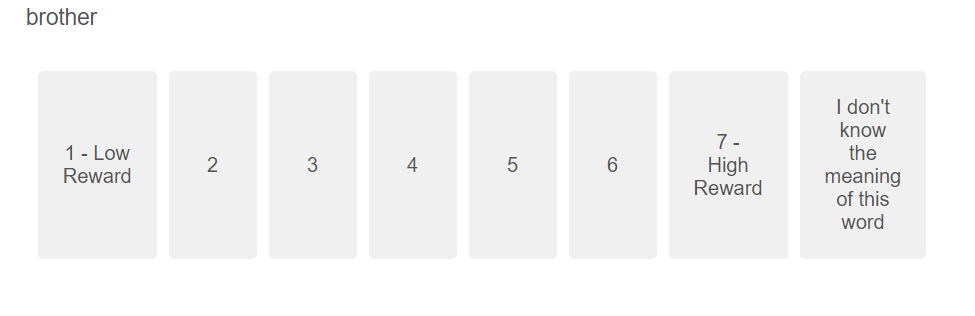
**

**Section S3. Pilot Stimuli**

**Table S3. Stimuli list (N=60) for the motivation measure**

| Concreteness | | |
| --- | --- | --- |
| Abstract | Intermediate | Concrete |
| *leisurely,* wit,  paradox,  *soulless,* repression, overwhelm | *new, jazzy*,  electron, cannibalism, migraine | butterfly, blueberry, whisk,  sludge, *thorny* |
| *clever,* allure,   fate, prefer, mood,   regret, *wrongful* | travel, revive, catalyst, clue, escape,  suspect, *greedy* | summerhouse, *sunny*,  auctioneer, bell, resume, mosquito, scream |
| aspire, possibility, knowledge,  evolve, motive,  *dangerous*, *unresolved,* animosity | *favorite*, explore, milestone,   speech, expand,   problem, rage | coffee, applaud, cash, festival,   climb, microphone   tornado, gunshot |

Note: The words were selected based on their concreteness and valence ratings, to span the entire continuum of each dimension. The valence of the stimuli is indicated by color: positive = green, neutral = yellow, negative = red. We included nouns, adjectives, and verbs: adjectives are in italics, while verbs are underlined.

**Table S4. Stimuli list (N=60) for the pleasure/reward measure**

| Concreteness | | |
| --- | --- | --- |
| Abstract | Intermediate | Concrete |
| infinity, innocence, *rhetorical,*  shame, hardship | dandy, humankind,  cater  *rancid,* reek | chipmunk, dolphin, trombone,  ulcer, *dead,* |
| awe, fascinate,  attribute, *spiritual*, *blasphemous*,  *smug,* deception | volunteer, *shiny,* nomination, work, *royal*,  spoils, domination | home, kitten, running  cellphone, acupuncture, contestant, tobacco, courtroom |
| hope, advantage, *heavenly*,  *excitable,* sarcasm,  supremacy, *selfish,* revenge | honeymoon*,* relax*, celebratory*,   *luxurious, carnal,* flattering,  rioting, cheat | kiss, honey, sailboat,   caress, caviar,   cocaine, cigarette |

Note: The words were selected based on their concreteness and valence ratings, to span the entire continuum of each dimension. The valence of the stimuli is indicated by color: positive = green, neutral = yellow, negative = red. We included nouns, adjectives, and verbs: adjectives are in italics, while verbs are underlined.

**Section S4. Motivation Rating Task Instructions**

‘Motivation’ is a driving force behind our behaviours, leading us to attain desirable goals. This study is concerned with objects, events, and interactions (and qualities of these things) that we experience and perceive as being motivating.

‘Motivation’ describes a sense of desire to achieve or obtain a goal. Therefore, there are many ways in which a word could refer to something ‘motivating’.

‘Motivation’ can be associated with internal factors (e.g., “gratitude”, “guilt”) or external factors (e.g., “bribe”, “cold”). Some words can refer to the motivation of interacting with others (e.g., “friends”, “presentation”), while others can refer to something you want to achieve (e.g., “status”, to “compete”). In contrast, there are things that are not at all motivating (e.g., “stone”). We are interested in exploring the extent to which words of all kinds are perceived as being related to ‘motivation’. Importantly, a word can relate to ‘motivation’ regardless of whether it refers to something positive or negative. For example, the word “tornado” should be given a high ‘motivation’ rating because it refers to something that will motivate a decision or action, despite having a negative connotation.

In this study, you are required to rate words based on the degree to which they have a meaning that is associated with ‘motivation’. Any word (e.g., “belief”) that in your estimation refers to something you perceive as being highly associated with ‘motivation’ should be given a high ‘motivation’ rating (at the upper end of the numerical scale). Any word (e.g., “duck”) that in your estimation refers to something that lacks an association with motivation should be given a low motivation rating (at the lower end of the scale). Any word that in your estimation refers to something that is not fundamentally motivating but has some motivating elements (e.g., ‘school’), or can be thought of as motivating in some circumstances (e.g., ‘audience’), should be given an intermediate motivation rating.

Please make your ‘motivation’ ratings using the 7-point scale. A value of 1 indicates a low ‘motivation’ rating, and a value of 7 indicates a high ‘motivation’ rating. The values ranging from 2 to 6 are intermediate ratings. Please feel free to use the whole range of values provided when making your ratings. Click on the rating that is most appropriate for each word. When making your ratings, try to be as accurate as possible, but do not spend too much time on any one word. If you are not familiar with a word’s meaning, please select ‘I don’t know the meaning of this word’.


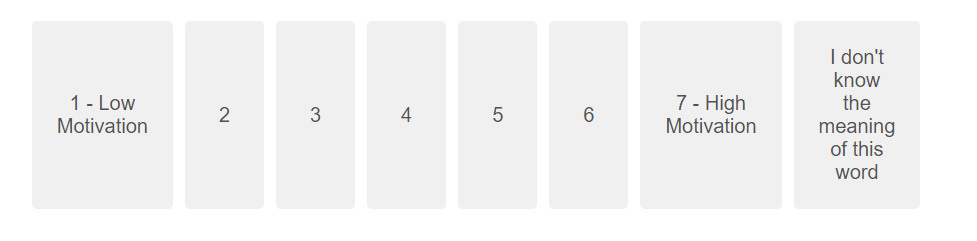


**Section S5. Additional Results for Hierarchical Regressions**

**Table S5.** Means, standard deviations and correlations of all variables for the regression analysis predicting performance in the English Lexicon Project Lexical Decision Task (N = 7,126)

| Variable | M | SD | 1 | 2 | 3 | 4 | 5 | 6 | 7 | 8 | 9 |
| --- | --- | --- | --- | --- | --- | --- | --- | --- | --- | --- | --- |
| 1. Length | 7.46 | 2.16 |  |  |  |  |  |  |  |  |  |
| 2. Frequency | 2.21 | 0.68 | -.39** |  |  |  |  |  |  |  |  |
| 3. Age of Acquisition | 9.27 | 2.50 | .35** | -.60** |  |  |  |  |  |  |  |
| 4. Motivation | 3.54 | 1.05 | .13** | .19** | .07** |  |  |  |  |  |  |
| 5. Concreteness | 3.12 | 0.98 | -.12** | .12** | -.39** | -.44** |  |  |  |  |  |
| 6. Valence | 5.12 | 1.32 | -.01 | .16** | -.22** | .24** | .08** |  |  |  |  |
| 7. Valence Extremity | 1.08 | 0.77 | .00 | .14** | -.14** | .25** | -.13** | -.08** |  |  |  |
| 8. Semantic Diversity | 1.60 | 0.31 | -.13** | .33** | -.15** | .29** | -.39** | .03* | .03* |  |  |
| 9. LDT zRT | -0.26 | 0.31 | .55** | -.61** | .57** | -.08** | -.15** | -.17** | -.07** | -.25** |  |
| 10. LDT Error Rate | 0.06 | 0.08 | -.05** | -.34** | .36** | -.16** | -.06** | -.12** | -.09** | -.15** | .51** |

*Note.* M and SD are used to represent mean and standard deviation, respectively. LDT = lexical decision task; zRT = standardized reaction times. * p < .05. ** p < .01.

**Table S6.** Means, standard deviations and correlations of all variables for the regression analysis predicting performance in the English Crowdsourcing Project Word Knowledge Task (N = 7,209)

| Variable | M | SD | 1 | 2 | 3 | 4 | 5 | 6 | 7 | 8 | 9 |
| --- | --- | --- | --- | --- | --- | --- | --- | --- | --- | --- | --- |
| 1. Length | 7.46 | 2.16 |  |  |  |  |  |  |  |  |  |
| 2. Frequency | 2.21 | 0.68 | -.39** |  |  |  |  |  |  |  |  |
| 3. Age of Acquisition | 9.28 | 2.50 | .35** | -.59** |  |  |  |  |  |  |  |
| 4. Motivation | 3.53 | 1.05 | .13** | .19** | .07** |  |  |  |  |  |  |
| 5. Concreteness | 3.12 | 0.97 | -.12** | .12** | -.39** | -.44** |  |  |  |  |  |
| 6. Valence | 5.12 | 1.32 | -.01 | .16** | -.22** | .24** | .08** |  |  |  |  |
| 7. Valence Extremity | 1.08 | 0.77 | .01 | .14** | -.14** | .26** | -.13** | -.09** |  |  |  |
| 8. Semantic Diversity | 1.59 | 0.31 | -.13** | .33** | -.15** | .29** | -.39** | .03* | .03* |  |  |
| 9. Recognition zRT | -0.53 | 0.11 | .42** | -.56** | .54** | -.11** | -.15** | -.18** | -.14** | -.23** |  |
| 10. Proportion Unknown | 0.01 | 0.02 | -.04** | -.35** | .37** | -.16** | -.06** | -.11** | -.12** | -.18** | .63** |

*Note.* M and SD are used to represent mean and standard deviation, respectively. zRT = standardized reaction times. * p < .05. ** p < .01.

**Table S7.** Means, standard deviations and correlations of all variables for the regression analysis predicting performance for concrete words in the Calgary Semantic Decision Task (N = 1958)

| Variable | M | SD | 1 | 2 | 3 | 4 | 5 | 6 | 7 | 8 | 9 |
| --- | --- | --- | --- | --- | --- | --- | --- | --- | --- | --- | --- |
| 1. Length | 0.00 | 1.00 |  |  |  |  |  |  |  |  |  |
| 2. Frequency | 0.00 | 1.00 | -.29** |  |  |  |  |  |  |  |  |
| 3. Age of Acquisition | 0.00 | 1.00 | .27** | -.48** |  |  |  |  |  |  |  |
| 4. Motivation | 0.00 | 1.00 | .17** | .25** | .06* |  |  |  |  |  |  |
| 5. Concreteness | 0.00 | 1.00 | -.09** | .13** | -.38** | -.35** |  |  |  |  |  |
| 6. Valence | 0.00 | 1.00 | .01 | .14** | -.31** | .02 | .21** |  |  |  |  |
| 7. Valence Extremity | 0.00 | 1.00 | -.02 | .16** | -.11** | .22** | -.03 | -.05* |  |  |  |
| 8. Semantic Diversity | 0.00 | 1.00 | -.15** | .39** | -.26** | .16** | -.09** | .02 | -.02 |  |  |
| 9. zRT | -0.21 | 0.44 | .21** | -.31** | .49** | .15** | -.60** | -.22** | -.04 | -.02 |  |
| 10. Errors | 0.12 | 0.14 | .07** | -.13** | .30** | .19** | -.55** | -.17** | -.01 | .09** | .74** |

*Note.* M and SD are used to represent mean and standard deviation, respectively. zRT = standardized reaction times. * p < .05. ** p < .01.

**Table S8.** Means, standard deviations and correlations of all variables for the regression analysis predicting performance for abstract words in the Calgary Semantic Decision Task (N = 2150)

| Variable | M | SD | 1 | 2 | 3 | 4 | 5 | 6 | 7 | 8 | 9 |
| --- | --- | --- | --- | --- | --- | --- | --- | --- | --- | --- | --- |
| 1. Length | 7.80 | 1.95 |  |  |  |  |  |  |  |  |  |
| 2. Frequency | 2.07 | 0.57 | -.33** |  |  |  |  |  |  |  |  |
| 3. Age of Acquisition | 10.33 | 2.06 | .23** | -.60** |  |  |  |  |  |  |  |
| 4. Motivation | 3.99 | 0.97 | .04 | .27** | -.16** |  |  |  |  |  |  |
| 5. Concreteness | 2.04 | 0.28 | -.12** | -.01 | -.09** | -.04 |  |  |  |  |  |
| 6. Valence | 5.08 | 1.45 | -.01 | .16** | -.12** | .52** | -.07** |  |  |  |  |
| 7. Valence Extremity | 1.22 | 0.78 | .02 | .18** | -.29** | .20** | -.02 | -.06** |  |  |  |
| 8. Semantic Diversity | 1.72 | 0.26 | -.06** | .28** | -.23** | .12** | -.11** | .06** | -.03 |  |  |
| 9. zRT | 0.09 | 0.33 | .09** | -.27** | .26** | -.15** | .25** | -.04* | -.20** | -.26** |  |
| 10. Errors | 0.13 | 0.14 | -.17** | -.01 | .03 | -.10** | .33** | -.00 | -.12** | -.24** | .53** |

*Note.* M and SD are used to represent mean and standard deviation, respectively. zRT = standardized reaction times. * p < .05. ** p < .01.

**Table S9.** Means, standard deviations and correlations of all variables for the regression analysis predicting performance in the Recognition Memory Task (N = 2,595)

| Variable | M | SD | 1 | 2 | 3 | 4 | 5 | 6 | 7 | 8 | 9 | 10 | 11 |
| --- | --- | --- | --- | --- | --- | --- | --- | --- | --- | --- | --- | --- | --- |
| 1. Length | 5.69 | 1.34 |  |  |  |  |  |  |  |  |  |  |  |
| 2. Frequency | 2.59 | 0.77 | -.28** |  |  |  |  |  |  |  |  |  |  |
| 3. OLD | 2.02 | 0.54 | .82** | -.31** |  |  |  |  |  |  |  |  |  |
| 4. Age of Acquisition | 8.34 | 2.60 | .29** | -.67** | .32** |  |  |  |  |  |  |  |  |
| 5. Motivation | 3.49 | 1.08 | .09** | .25** | .06** | .04* |  |  |  |  |  |  |  |
| 6. Concreteness | 3.26 | 0.98 | -.09** | .09** | -.10** | -.40** | -.50** |  |  |  |  |  |  |
| 7. Valence | 5.18 | 1.29 | -.00 | .24** | -.03 | -.26** | .21** | .10** |  |  |  |  |  |
| 8. Valence Extremity | 1.06 | 0.76 | -.02 | .20** | .02 | -.17** | .28** | -.08** | -.02 |  |  |  |  |
| 9. Semantic Diversity | 1.68 | 0.29 | -.06** | .37** | -.12** | -.15** | .35** | -.40** | .07** | -.01 |  |  |  |
| 10. Hits | 0.72 | 0.09 | .06** | -.42** | .13** | .26** | -.24** | .20** | -.15** | .01 | -.43** |  |  |
| 11. FalseAlarms | 0.22 | 0.10 | .14** | -.11** | -.03 | .16** | .20** | -.23** | -.08** | -.09** | .23** | .10** |  |
| 12. Hit_min_FA | 0.50 | 0.13 | -.06** | -.22** | .12** | .06** | -.33** | .33** | -.05* | .08** | -.48** | .64** | -.70** |

*Note.* M and SD are used to represent mean and standard deviation, respectively. Hit_min_FA = difference between hits and false alarms. * p < .05. ** p < .01.

**Table S10.** *Regression coefficients for item-level regression analyses of English Lexicon Project Lexical Decision Task RTs and error rates (N = 7126)*

| Predictor | zRT |  |  |  |  |  |  | Error rates | |  |  |  |  |  |
| --- | --- | --- | --- | --- | --- | --- | --- | --- | --- | --- | --- | --- | --- | --- |
|  | b | SE | t | p | sr2 | R2 | ∆R2 | b | SE | t | p | sr2 | R2 | ∆R2 |
| Step1 |  |  |  |  |  | 0.53 | 0.53 |  |  |  |  |  | 0.2 | 0.2 |
| Intercept | -0.261 | 0.003 | -102.64 | 0 |  |  |  | 0.06 | 0.001 | 71.83 | 0 |  |  |  |
| Length | 0.047 | 0.001 | 36.51 | 0 | 0.089 |  |  | -0.009 | 0 | -21.97 | 0 | 0.054 |  |  |
| Frequency | -0.146 | 0.005 | -30.42 | 0 | 0.062 |  |  | -0.031 | 0.002 | -19.5 | 0 | 0.042 |  |  |
| Age of Acquisition | 0.034 | 0.001 | 26.27 | *** | 0.046 |  |  | 0.009 | 0 | 21.57 | *** | 0.052 |  |  |
| Step2 |  |  |  |  |  | 0.54 | 0.01 |  |  |  |  |  | 0.22 | 0.01 |
| Intercept | -0.261 | 0.003 | -103.78 | *** |  |  |  | 0.06 | 0.001 | 72.33 | *** |  |  |  |
| Length | 0.05 | 0.001 | 37.87 | *** | 0.093 |  |  | -0.009 | 0 | -19.93 | *** | 0.044 |  |  |
| Frequency | -0.122 | 0.005 | -23.47 | *** | 0.036 |  |  | -0.025 | 0.002 | -14.33 | *** | 0.023 |  |  |
| Age of Acquisition | 0.036 | 0.001 | 25.41 | *** | 0.042 |  |  | 0.01 | 0 | 21.46 | *** | 0.051 |  |  |
| Concreteness | -0.008 | 0.004 | -2.2 | * | 0 |  |  | 0 | 0.001 | 0.28 | 0.777 | 0 |  |  |
| Valence Extremity | 0.01 | 0.003 | 2.93 | ** | 0.001 |  |  | 0.001 | 0.001 | 0.6 | 0.548 | 0 |  |  |
| Semantic Diversity | -0.058 | 0.01 | -5.92 | *** | 0.002 |  |  | -0.01 | 0.003 | -2.92 | ** | 0.001 |  |  |
| Motivation | -0.028 | 0.003 | -9.62 | *** | 0.006 |  |  | -0.007 | 0.001 | -7.63 | *** | 0.006 |  |  |

*Note*. b represents the unstandardized beta coefficients. SE represents the standard error of the beta coefficients. sr^2^ represents the semi-partial correlation squared. zRTs refers to the standardized reaction times. **p*<.05; ***p*<.01; ****p*<.001

**Table S11.** *Regression coefficients for item-level regression analyses predicting English Crowdsourcing Project Word Knowledge Task reaction times and proportion unknown (N = 7209)*

| Predictor | zRT |  |  |  |  |  |  | Proportion unknown | | | |  |  |  |
| --- | --- | --- | --- | --- | --- | --- | --- | --- | --- | --- | --- | --- | --- | --- |
|  | b | SE | t | p | sr2 | R2 | ∆R2 | b | SE | t | p | sr2 | R2 | ∆R2 |
| Step1 |  |  |  |  |  | 0.42 | 0.42 |  |  |  |  |  | 0.22 | 0.22 |
| Intercept | -0.533 | 0.001 | -517.08 | *** |  |  |  | 0.012 | 0 | 71.05 | *** |  |  |  |
| Length | 0.011 | 0.001 | 20.36 | *** | 0.034 |  |  | -0.002 | 0 | -21.96 | *** | 0.052 |  |  |
| Frequency | -0.054 | 0.002 | -27.73 | *** | 0.062 |  |  | -0.007 | 0 | -20.54 | *** | 0.046 |  |  |
| Age of Acquisition | 0.013 | 0.001 | 24.69 | *** | 0.049 |  |  | 0.002 | 0 | 22.69 | *** | 0.056 |  |  |
| Step2 |  |  |  |  |  | 0.43 | 0.01 |  |  |  |  |  | 0.23 | 0.01 |
| Intercept | -0.533 | 0.001 | -523.78 | *** |  |  |  | 0.012 | 0 | 71.73 | *** |  |  |  |
| Length | 0.012 | 0.001 | 22.71 | *** | 0.041 |  |  | -0.002 | 0 | -19.85 | *** | 0.042 |  |  |
| Frequency | -0.043 | 0.002 | -20.73 | *** | 0.034 |  |  | -0.005 | 0 | -14.47 | *** | 0.022 |  |  |
| Age of Acquisition | 0.013 | 0.001 | 22.42 | *** | 0.04 |  |  | 0.002 | 0 | 21.95 | *** | 0.051 |  |  |
| Concreteness | -0.007 | 0.001 | -4.61 | *** | 0.002 |  |  | 0 | 0 | -0.81 | 0.415 | 0 |  |  |
| Valence Extremity | -0.006 | 0.001 | -4.58 | *** | 0.002 |  |  | 0 | 0 | -1.72 | 0.085 | 0 |  |  |
| Semantic Diversity | -0.021 | 0.004 | -5.27 | *** | 0.002 |  |  | -0.004 | 0.001 | -5.78 | *** | 0.004 |  |  |
| Motivation | -0.012 | 0.001 | -10.41 | *** | 0.009 |  |  | -0.002 | 0 | -7.55 | *** | 0.006 |  |  |

*Note*. b represents the unstandardized beta coefficients. SE represents the standard error of the beta coefficients. sr^2^ represents the semi-partial correlation squared. zRTs refers to the standardized reaction times. **p*<.05; ***p*<.01; ****p*<.001

**Table S12.** *Regression coefficients from item-level regression analyses predicting Calgary Semantic Decision Task*  *reaction times and error rates for concrete words (N = 1958)*

| Predictor | zRT |  |  |  |  |  |  | Error rate | | |  |  |  |  |
| --- | --- | --- | --- | --- | --- | --- | --- | --- | --- | --- | --- | --- | --- | --- |
|  | b | SE | t | p | sr2 | R2 | ∆R2 | b | SE | t | p | sr2 | R2 | ∆R2 |
| Step1 |  |  |  |  |  | 0.25 | 0.25 |  |  |  |  |  | 0.09 | 0.09 |
| Intercept | -0.208 | 0.009 | -24.14 | *** |  |  |  | 0.117 | 0.003 | 38.56 | *** |  |  |  |
| Length | 0.03 | 0.009 | 3.26 | ** | 0.004 |  |  | -0.002 | 0.003 | -0.74 | 0.457 | 0 |  |  |
| Frequency | -0.036 | 0.01 | -3.57 | *** | 0.005 |  |  | 0.001 | 0.004 | 0.38 | 0.702 | 0 |  |  |
| Age of Acquisition | 0.193 | 0.01 | 19.4 | *** | 0.143 |  |  | 0.044 | 0.004 | 12.42 | *** | 0.072 |  |  |
| Step2 |  |  |  |  |  | 0.46 | 0.21 |  |  |  |  |  | 0.32 | 0.23 |
| Intercept | -0.208 | 0.007 | -28.44 | *** |  |  |  | 0.117 | 0.003 | 44.72 | *** |  |  |  |
| Length | 0.034 | 0.008 | 4.21 | *** | 0.005 |  |  | -0.002 | 0.003 | -0.81 | 0.419 | 0 |  |  |
| Frequency | -0.054 | 0.009 | -5.73 | *** | 0.009 |  |  | -0.008 | 0.003 | -2.38 | * | 0.002 |  |  |
| Age of Acquisition | 0.111 | 0.009 | 12.05 | *** | 0.04 |  |  | 0.016 | 0.003 | 4.91 | *** | 0.008 |  |  |
| Concreteness | -0.214 | 0.009 | -24.83 | *** | 0.169 |  |  | -0.069 | 0.003 | -22.42 | *** | 0.174 |  |  |
| Valence Extremity | 0 | 0.008 | -0.03 | 0.975 | 0 |  |  | -0.001 | 0.003 | -0.35 | 0.727 | 0 |  |  |
| Semantic Diversity | 0.029 | 0.008 | 3.59 | *** | 0.004 |  |  | 0.013 | 0.003 | 4.46 | *** | 0.007 |  |  |
| Motivation | -0.011 | 0.009 | -1.24 | 0.216 | 0 |  |  | 0.002 | 0.003 | 0.69 | 0.493 | 0 |  |  |

*Note*. b represents the unstandardized beta coefficients. SE represents the standard error of the beta coefficients. sr^2^ represents the semi-partial correlation squared. zRTs refers to the standardized reaction times. **p*<.05; ***p*<.01; ****p*<.001

**Table S13.** *Regression coefficients from item-level regression analyses predicting Calgary Semantic Decision Task* *reaction times and error rates for abstract words (N = 2150)*

| Predictor | zRT | | | | | | | Error rate | | | | | | |
| --- | --- | --- | --- | --- | --- | --- | --- | --- | --- | --- | --- | --- | --- | --- |
|  | b | SE | t | p | sr2 | R2 | ∆R2 | b | SE | t | p | sr2 | R2 | ∆R2 |
| Step1 |  |  |  |  |  | 0.08 | 0.08 |  |  |  |  |  | 0.03 | 0.03 |
| Intercept | 0.056 | 0.074 | 0.75 | 0.451 |  |  |  | 0.225 | 0.032 | 7.08 | 0 |  |  |  |
| Length | -0.001 | 0.004 | -0.18 | 0.855 | 0 |  |  | -0.013 | 0.002 | -8.51 | 0 | 0.033 |  |  |
| Frequency | -0.101 | 0.015 | -6.6 | 0 | 0.019 |  |  | -0.01 | 0.007 | -1.54 | 0.124 | 0.001 |  |  |
| Age of Acquisition | 0.024 | 0.004 | 5.95 | 0 | 0.015 |  |  | 0.003 | 0.002 | 1.71 | 0.087 | 0.001 |  |  |
| Step2 |  |  |  |  |  | 0.2 | 0.12 |  |  |  |  |  | 0.19 | 0.16 |
| Intercept | -0.169 | 0.106 | -1.6 | 0.109 |  |  |  | 0.115 | 0.044 | 2.6 | 0.009 |  |  |  |
| Length | 0.009 | 0.003 | 2.62 | 0.009 | 0.003 |  |  | -0.009 | 0.001 | -6.04 | 0 | 0.014 |  |  |
| Frequency | -0.047 | 0.015 | -3.14 | 0.002 | 0.004 |  |  | 0.017 | 0.006 | 2.68 | 0.008 | 0.003 |  |  |
| Age of Acquisition | 0.02 | 0.004 | 5.13 | 0 | 0.01 |  |  | 0.002 | 0.002 | 1.15 | 0.251 | 0 |  |  |
| Concreteness | 0.288 | 0.023 | 12.51 | 0 | 0.058 |  |  | 0.142 | 0.01 | 14.71 | 0 | 0.081 |  |  |
| Valence Extremity | -0.058 | 0.009 | -6.69 | 0 | 0.017 |  |  | -0.02 | 0.004 | -5.51 | 0 | 0.011 |  |  |
| Semantic Diversity | -0.219 | 0.026 | -8.53 | 0 | 0.027 |  |  | -0.12 | 0.011 | -11.14 | 0 | 0.047 |  |  |
| Motivation | -0.015 | 0.007 | -2.19 | 0.029 | 0.002 |  |  | -0.007 | 0.003 | -2.32 | 0.021 | 0.002 |  |  |

*Note*. b represents the unstandardized beta coefficients. SE represents the standard error of the beta coefficients. sr^2^ represents the semi-partial correlation squared. zRTs refers to the standardized reaction times. **p*<.05; ***p*<.01; ****p*<.001

**Table S14.** *Regression coefficients from item-level regression analyses predicting Recognition Memory Task hits and false alarms rates (N = 2595)*

| Predictor | Hits |  |  |  |  |  |  | FA |  |  |  |  |  |  |
| --- | --- | --- | --- | --- | --- | --- | --- | --- | --- | --- | --- | --- | --- | --- |
|  | b | SE | t | p | sr2 | R2 | ∆R2 | b | SE | t | p | sr2 | R2 | ∆R2 |
| Step1 |  |  |  |  |  | 0.18 | 0.18 |  |  |  |  |  | 0.11 | 0.11 |
| Intercept | 0.719 | 0.002 | 433.3 | *** |  |  |  | 0.218 | 0.002 | 117.84 | *** |  |  |  |
| Length | -0.016 | 0.003 | -5.45 | *** | 0.009 |  |  | 0.049 | 0.003 | 15.02 | *** | 0.077 |  |  |
| Frequency | -0.041 | 0.002 | -18.09 | *** | 0.103 |  |  | 0 | 0.003 | -0.08 | 0.939 | 0 |  |  |
| OLD | 0.013 | 0.003 | 4.5 | *** | 0.006 |  |  | -0.049 | 0.003 | -14.93 | *** | 0.076 |  |  |
| Age of Acquisition | -0.002 | 0.002 | -1 | 0.317 | 0 |  |  | 0.018 | 0.003 | 7.12 | *** | 0.017 |  |  |
| Step2 |  |  |  |  |  | 0.29 | 0.11 |  |  |  |  |  | 0.19 | 0.08 |
| Intercept | 0.719 | 0.002 | 466.51 | *** |  |  |  | 0.218 | 0.002 | 123.78 | *** |  |  |  |
| Length | -0.011 | 0.003 | -4.07 | *** | 0.004 |  |  | 0.043 | 0.003 | 13.55 | *** | 0.057 |  |  |
| Frequency | -0.025 | 0.002 | -10.8 | *** | 0.032 |  |  | -0.018 | 0.003 | -6.8 | *** | 0.014 |  |  |
| OLD | 0.008 | 0.003 | 2.97 | ** | 0.002 |  |  | -0.045 | 0.003 | -13.97 | *** | 0.061 |  |  |
| Age of Acquisition | 0.015 | 0.002 | 6.13 | *** | 0.01 |  |  | 0.005 | 0.003 | 1.87 | 0.062 | 0.001 |  |  |
| Concreteness | 0.019 | 0.002 | 8.54 | *** | 0.02 |  |  | -0.005 | 0.002 | -1.97 | * | 0.001 |  |  |
| Valence Extremity | 0.01 | 0.002 | 6.21 | *** | 0.01 |  |  | -0.008 | 0.002 | -4.15 | *** | 0.005 |  |  |
| Semantic Diversity | -0.019 | 0.002 | -9.87 | *** | 0.026 |  |  | 0.02 | 0.002 | 9.1 | *** | 0.026 |  |  |
| Motivation | -0.004 | 0.002 | -1.81 | 0.071 | 0.001 |  |  | 0.016 | 0.002 | 6.99 | *** | 0.015 |  |  |

*Note*. b represents the unstandardized beta coefficients. SE represents the standard error of the beta coefficients. sr^2^ represents the semi-partial correlation squared. **p*<.05; ***p*<.01; ****p*<.001

**Table S15.** *Regression coefficients from item-level regression analyses predicting Recognition Memory Task hits minus false alarms (N = 2595).*

| Predictor | H - FA |  |  |  |  |  |  |
| --- | --- | --- | --- | --- | --- | --- | --- |
|  | b | SE | t | p | sr2 | R2 | ∆R2 |
| Step1 |  |  |  |  |  | 0.14 | 0.14 |
| Intercept | 0.501 | 0.002 | 211.92 | *** |  |  |  |
| Length | -0.065 | 0.004 | -15.49 | *** | 0.079 |  |  |
| Frequency | -0.041 | 0.003 | -12.63 | *** | 0.053 |  |  |
| OLD | 0.063 | 0.004 | 14.74 | *** | 0.072 |  |  |
| Age of Acquisition | -0.02 | 0.003 | -6.27 | *** | 0.013 |  |  |
| Step2 |  |  |  |  |  | 0.35 | 0.21 |
| Intercept | 0.501 | 0.002 | 243.28 | *** |  |  |  |
| Length | -0.054 | 0.004 | -14.54 | *** | 0.053 |  |  |
| Frequency | -0.007 | 0.003 | -2.26 | * | 0.001 |  |  |
| OLD | 0.052 | 0.004 | 14.05 | *** | 0.05 |  |  |
| Age of Acquisition | 0.01 | 0.003 | 2.98 | ** | 0.002 |  |  |
| Concreteness | 0.024 | 0.003 | 8.08 | *** | 0.016 |  |  |
| Valence | 0.018 | 0.002 | 8.14 | *** | 0.017 |  |  |
| Semantic Diversity | -0.039 | 0.003 | -15.19 | *** | 0.058 |  |  |
| Motivation | -0.019 | 0.003 | -7.27 | *** | 0.013 |  |  |

*Note*. b represents the unstandardized beta coefficients. SE represents the standard error of the beta coefficients. sr^2^ represents the semi-partial correlation squared. **p*<.05; ***p*<.01; ****p*<.001

**Section S6. Scatterplots of the Correlations of Motivation with other Lexical and Semantic Dimensions**


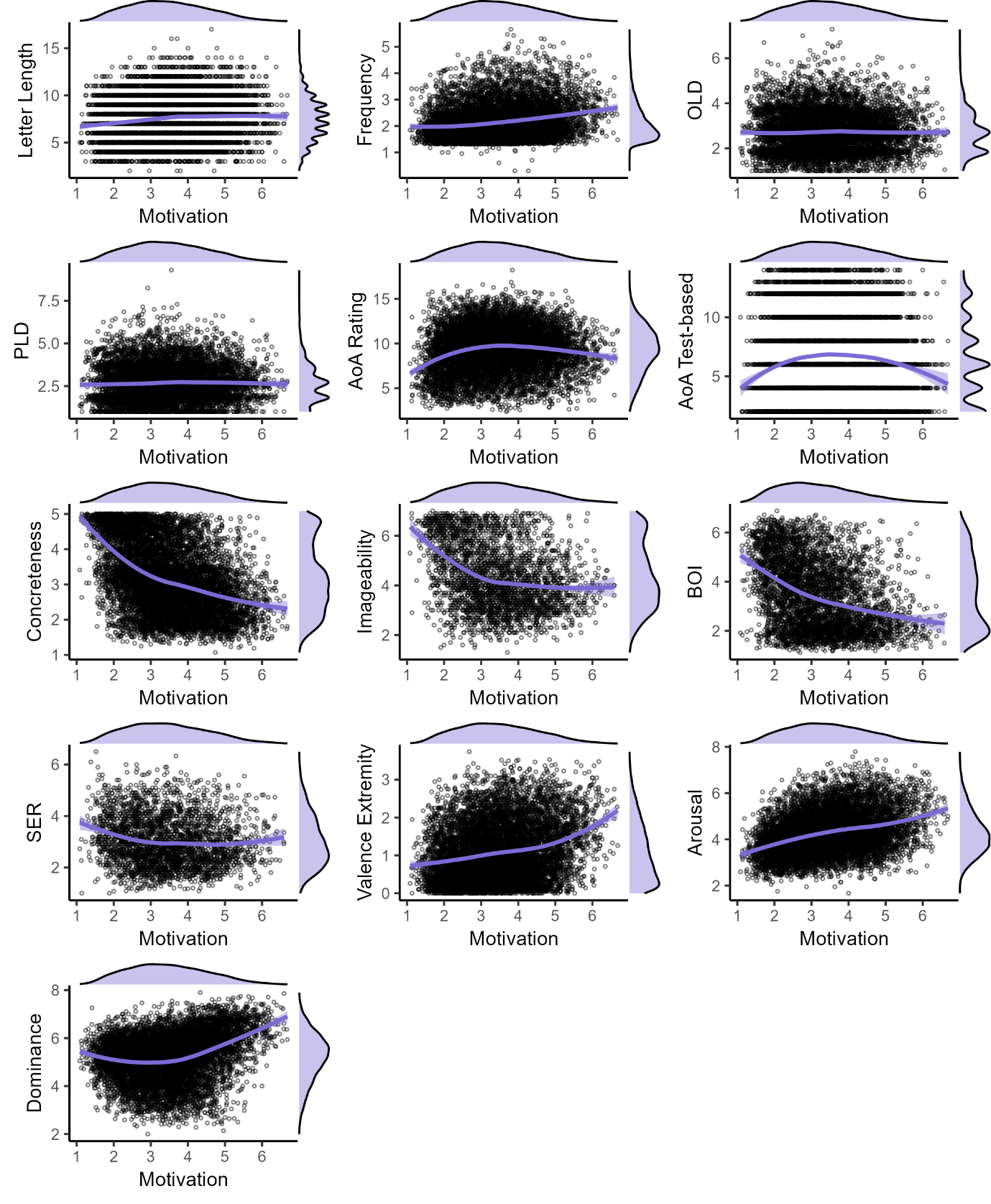


**Figure S1.** Scatterplots illustrating the relationships (highlighted by the loess line in purple) between mean motivation ratings and lexical-semantic dimensions. The density distributions of motivation and the variables of interest are plotted on the top and right of the graphs, respectively.

**Section S7. Composite Measure**

**Table S16.** *Regression coefficients from item-level regression analyses predicting English Lexicon Project Lexical Decision Task reaction times and error rates (N = 6860)*

|  | zRT | | | | | | | Error rate | | | | | | |
| --- | --- | --- | --- | --- | --- | --- | --- | --- | --- | --- | --- | --- | --- | --- |
| Predictor | b | SE | t | p | sr2 | R2 | ∆R2 | b | SE | t | p | sr2 | R2 | ∆R2 |
|  |  |  |  |  |  | 0.54 | 0.01 |  |  |  |  |  | 0.22 | 0.02 |
| Intercept | -0.261 | 0.003 | -103.67 | *** |  |  |  | 0.06 | 0.001 | 72.33 | *** |  |  |  |
| Length | 0.05 | 0.001 | 37.79 | *** | 0.093 |  |  | -0.009 | 0 | -19.91 | *** | 0.044 |  |  |
| Frequency | -0.122 | 0.005 | -23.45 | *** | 0.036 |  |  | -0.025 | 0.002 | -14.29 | *** | 0.023 |  |  |
| Age of Acquisition | 0.035 | 0.001 | 25.08 | *** | 0.041 |  |  | 0.01 | 0 | 21.55 | *** | 0.051 |  |  |
| Concreteness | -0.009 | 0.003 | -2.53 | * | 0 |  |  | 0 | 0.001 | 0.14 | 0.89 | 0 |  |  |
| Semantic Diversity | -0.064 | 0.01 | -6.57 | *** | 0.003 |  |  | -0.01 | 0.003 | -3.18 | ** | 0.001 |  |  |
| Minkowski3 | -0.025 | 0.003 | -8.61 | *** | 0.005 |  |  | -0.007 | 0.001 | -7.54 | *** | 0.006 |  |  |
|  |  |  |  |  |  | 0.53 | 0 |  |  |  |  |  | 0.21 | 0.01 |
| Intercept | -0.261 | 0.003 | -103.59 | *** |  |  |  | 0.06 | 0.001 | 72.3 | *** |  |  |  |
| Length | 0.049 | 0.001 | 37.63 | *** | 0.092 |  |  | -0.009 | 0 | -19.99 | *** | 0.044 |  |  |
| Frequency | -0.123 | 0.005 | -23.65 | *** | 0.037 |  |  | -0.025 | 0.002 | -14.4 | *** | 0.023 |  |  |
| Age of Acquisition | 0.035 | 0.001 | 24.85 | *** | 0.04 |  |  | 0.01 | 0 | 21.36 | *** | 0.05 |  |  |
| Concreteness | -0.008 | 0.004 | -2.35 | * | 0 |  |  | 0 | 0.001 | 0.18 | 0.856 | 0 |  |  |
| Semantic Diversity | -0.066 | 0.01 | -6.75 | *** | 0.003 |  |  | -0.011 | 0.003 | -3.34 | ** | 0.001 |  |  |
| Euclidean | -0.022 | 0.003 | -7.95 | *** | 0.004 |  |  | -0.007 | 0.001 | -7.24 | *** | 0.006 |  |  |
|  |  |  |  |  |  | 0.53 | 0 |  |  |  |  |  | 0.21 | 0.01 |
| Intercept | -0.261 | 0.003 | -103.38 | *** |  |  |  | 0.06 | 0.001 | 72.21 | *** |  |  |  |
| Length | 0.049 | 0.001 | 37.13 | *** | 0.09 |  |  | -0.009 | 0 | -20.49 | *** | 0.046 |  |  |
| Frequency | -0.127 | 0.005 | -24.58 | *** | 0.04 |  |  | -0.026 | 0.002 | -15.07 | *** | 0.025 |  |  |
| Age of Acquisition | 0.034 | 0.001 | 24.38 | *** | 0.039 |  |  | 0.01 | 0 | 20.9 | *** | 0.048 |  |  |
| Concreteness | -0.005 | 0.004 | -1.46 | 0.144 | 0 |  |  | 0.001 | 0.001 | 0.75 | 0.454 | 0 |  |  |
| Semantic Diversity | -0.069 | 0.01 | -7.05 | *** | 0.003 |  |  | -0.012 | 0.003 | -3.65 | *** | 0.001 |  |  |
| Sum Strength | -0.012 | 0.002 | -5.81 | *** | 0.002 |  |  | -0.004 | 0.001 | -5.91 | *** | 0.004 |  |  |

*Note*. b represents the unstandardized beta coefficients. SE represents the standard error of the beta coefficients. sr^2^ represents the semi-partial correlation squared. **p*<.05; ***p*<.01; ****p*<.001

**Table S17.** *Regression coefficients from item-level regression analyses predicting English Crowdsourcing Project Word Knowledge Task reaction times and proportion unknown (N = 6943).*

|  | zRT | | | | | | | Proportion Unknown | | | | | | |
| --- | --- | --- | --- | --- | --- | --- | --- | --- | --- | --- | --- | --- | --- | --- |
| Predictor | b | SE | t | p | sr2 | R2 | ∆R2 | b | SE | t | p | sr2 | R2 | ∆R2 |
|  |  |  |  |  |  | 0.43 | 0.01 |  |  |  |  |  | 0.23 | 0.01 |
| Intercept | -0.533 | 0.001 | -523.4 | *** |  |  |  | 0.012 | 0 | 71.74 | *** |  |  |  |
| Length | 0.012 | 0.001 | 22.65 | *** | 0.041 |  |  | -0.002 | 0 | -19.83 | *** | 0.042 |  |  |
| Frequency | -0.043 | 0.002 | -20.73 | *** | 0.034 |  |  | -0.005 | 0 | -14.43 | *** | 0.022 |  |  |
| Age of Acquisition | 0.013 | 0.001 | 23.36 | *** | 0.043 |  |  | 0.002 | 0 | 22.47 | *** | 0.054 |  |  |
| Concreteness | -0.006 | 0.001 | -4.23 | *** | 0.001 |  |  | 0 | 0 | -0.76 | 0.446 | 0 |  |  |
| Semantic Diversity | -0.019 | 0.004 | -4.89 | *** | 0.002 |  |  | -0.004 | 0.001 | -5.75 | *** | 0.004 |  |  |
| Minkowski3 | -0.014 | 0.001 | -11.81 | *** | 0.011 |  |  | -0.002 | 0 | -8.25 | *** | 0.007 |  |  |
|  |  |  |  |  |  | 0.43 | 0.01 |  |  |  |  |  | 0.23 | 0.01 |
| Intercept | -0.533 | 0.001 | -523.66 | *** |  |  |  | 0.012 | 0 | 71.74 | *** |  |  |  |
| Length | 0.012 | 0.001 | 22.72 | *** | 0.041 |  |  | -0.002 | 0 | -19.84 | *** | 0.042 |  |  |
| Frequency | -0.043 | 0.002 | -20.69 | *** | 0.034 |  |  | -0.005 | 0 | -14.45 | *** | 0.022 |  |  |
| Age of Acquisition | 0.013 | 0.001 | 23.07 | *** | 0.042 |  |  | 0.002 | 0 | 22.26 | *** | 0.053 |  |  |
| Concreteness | -0.006 | 0.001 | -4.46 | *** | 0.002 |  |  | 0 | 0 | -0.84 | 0.4 | 0 |  |  |
| Semantic Diversity | -0.02 | 0.004 | -5.13 | *** | 0.002 |  |  | -0.004 | 0.001 | -5.92 | *** | 0.004 |  |  |
| Euclidean | -0.013 | 0.001 | -12.12 | *** | 0.012 |  |  | -0.002 | 0 | -8.25 | *** | 0.007 |  |  |
|  |  |  |  |  |  | 0.43 | 0.01 |  |  |  |  |  | 0.23 | 0.01 |
| Intercept | -0.533 | 0.001 | -523.6 | *** |  |  |  | 0.012 | 0 | 71.69 | *** |  |  |  |
| Length | 0.012 | 0.001 | 22.52 | *** | 0.04 |  |  | -0.002 | 0 | -20.25 | *** | 0.044 |  |  |
| Frequency | -0.044 | 0.002 | -21.25 | *** | 0.036 |  |  | -0.005 | 0 | -14.99 | *** | 0.024 |  |  |
| Age of Acquisition | 0.013 | 0.001 | 22.23 | *** | 0.039 |  |  | 0.002 | 0 | 21.7 | *** | 0.05 |  |  |
| Concreteness | -0.006 | 0.001 | -4.35 | *** | 0.001 |  |  | 0 | 0 | -0.52 | 0.603 | 0 |  |  |
| Semantic Diversity | -0.023 | 0.004 | -5.74 | *** | 0.003 |  |  | -0.004 | 0.001 | -6.3 | *** | 0.004 |  |  |
| Sum Strength | -0.01 | 0.001 | -12.05 | *** | 0.011 |  |  | -0.001 | 0 | -7.56 | *** | 0.006 |  |  |

*Note*. b represents the unstandardized beta coefficients. SE represents the standard error of the beta coefficients. sr^2^ represents the semi-partial correlation squared. **p*<.05; ***p*<.01; ****p*<.001

**Table S18.** *Regression coefficients from item-level regression analyses predicting* *Calgary Semantic Decision Task reaction times and error rates for concrete words*

|  | zRT | | | | | | | Error rate | | | | | | |
| --- | --- | --- | --- | --- | --- | --- | --- | --- | --- | --- | --- | --- | --- | --- |
| Predictor | b | SE | t | p | sr2 | R2 | ∆R2 | b | SE | t | p | sr2 | R2 | ∆R2 |
|  |  |  |  |  |  | 0.46 | 0.21 |  |  |  |  |  | 0.32 | 0.23 |
| Intercept | -0.208 | 0.007 | -28.45 | *** |  |  |  | 0.117 | 0.003 | 44.73 | *** |  |  |  |
| Length | 0.034 | 0.008 | 4.21 | *** | 0.005 |  |  | -0.002 | 0.003 | -0.8 | 0.421 | 0 |  |  |
| Frequency | -0.054 | 0.009 | -5.73 | *** | 0.009 |  |  | -0.008 | 0.003 | -2.42 | * | 0.002 |  |  |
| Age of Acquisition | 0.11 | 0.009 | 12.1 | *** | 0.04 |  |  | 0.016 | 0.003 | 4.96 | *** | 0.009 |  |  |
| Concreteness | -0.214 | 0.009 | -24.87 | *** | 0.17 |  |  | -0.069 | 0.003 | -22.45 | *** | 0.174 |  |  |
| Semantic Diversity | 0.029 | 0.008 | 3.6 | *** | 0.004 |  |  | 0.013 | 0.003 | 4.54 | *** | 0.007 |  |  |
| Minkowski3 | -0.011 | 0.009 | -1.27 | 0.203 | 0 |  |  | 0.002 | 0.003 | 0.65 | 0.514 | 0 |  |  |
|  |  |  |  |  |  | 0.46 | 0.21 |  |  |  |  |  | 0.32 | 0.23 |
| Intercept | -0.208 | 0.007 | -28.45 | *** |  |  |  | 0.117 | 0.003 | 44.73 | *** |  |  |  |
| Length | 0.033 | 0.008 | 4.19 | *** | 0.005 |  |  | -0.002 | 0.003 | -0.8 | 0.426 | 0 |  |  |
| Frequency | -0.055 | 0.009 | -5.75 | *** | 0.009 |  |  | -0.008 | 0.003 | -2.41 | ** | 0.002 |  |  |
| Age of Acquisition | 0.11 | 0.009 | 12.08 | *** | 0.04 |  |  | 0.016 | 0.003 | 4.97 | *** | 0.009 |  |  |
| Concreteness | -0.214 | 0.009 | -24.91 | *** | 0.17 |  |  | -0.069 | 0.003 | -22.54 | *** | 0.176 |  |  |
| Semantic Diversity | 0.029 | 0.008 | 3.58 | *** | 0.004 |  |  | 0.013 | 0.003 | 4.55 | *** | 0.007 |  |  |
| Euclidean | -0.01 | 0.008 | -1.2 | 0.232 | 0 |  |  | 0.002 | 0.003 | 0.64 | 0.524 | 0 |  |  |
|  |  |  |  |  |  | 0.46 | 0.21 |  |  |  |  |  | 0.32 | 0.23 |
| Intercept | -0.208 | 0.007 | -28.45 | *** |  |  |  | 0.117 | 0.003 | 44.73 | *** |  |  |  |
| Length | 0.033 | 0.008 | 4.14 | *** | 0.005 |  |  | -0.002 | 0.003 | -0.71 | 0.479 | 0 |  |  |
| Frequency | -0.055 | 0.009 | -5.89 | *** | 0.01 |  |  | -0.008 | 0.003 | -2.31 | * | 0.002 |  |  |
| Age of Acquisition | 0.11 | 0.009 | 12.04 | *** | 0.04 |  |  | 0.016 | 0.003 | 4.99 | *** | 0.009 |  |  |
| Concreteness | -0.213 | 0.008 | -25.22 | *** | 0.174 |  |  | -0.07 | 0.003 | -23.05 | *** | 0.184 |  |  |
| Semantic Diversity | 0.029 | 0.008 | 3.53 | *** | 0.003 |  |  | 0.013 | 0.003 | 4.56 | *** | 0.007 |  |  |
| Sum Strength | -0.008 | 0.008 | -1.03 | 0.301 | 0 |  |  | 0.001 | 0.003 | 0.3 | 0.766 | 0 |  |  |

*Note*. b represents the unstandardized beta coefficients. SE represents the standard error of the beta coefficients. sr^2^ represents the semi-partial correlation squared. **p*<.05; ***p*<.01; ****p*<.001

**Table S19.** *Regression coefficients from item-level regression analyses predicting* *Calgary Semantic Decision Task reaction times and error rates for abstract words*

|  | zRT | | | | | | | Error rate | | | | | | |
| --- | --- | --- | --- | --- | --- | --- | --- | --- | --- | --- | --- | --- | --- | --- |
| Predictor | b | SE | t | p | sr2 | R2 | ∆R2 | b | SE | t | p | sr2 | R2 | ∆R2 |
|  |  |  |  |  |  | 0.19 | 0.11 |  |  |  |  |  | 0.18 | 0.15 |
| Intercept | -0.295 | 0.104 | -2.82 | ** |  |  |  | 0.072 | 0.044 | 1.66 | 0.098 |  |  |  |
| Length | 0.008 | 0.004 | 2.31 | * | 0.002 |  |  | -0.009 | 0.001 | -6.26 | *** | 0.015 |  |  |
| Frequency | -0.044 | 0.015 | -2.93 | ** | 0.003 |  |  | 0.018 | 0.006 | 2.8 | ** | 0.003 |  |  |
| Age of Acquisition | 0.027 | 0.004 | 6.9 | *** | 0.018 |  |  | 0.004 | 0.002 | 2.52 | * | 0.002 |  |  |
| Concreteness | 0.295 | 0.023 | 12.74 | *** | 0.061 |  |  | 0.144 | 0.01 | 14.9 | *** | 0.085 |  |  |
| Semantic Diversity | -0.198 | 0.026 | -7.71 | *** | 0.022 |  |  | -0.113 | 0.011 | -10.51 | *** | 0.042 |  |  |
| Minkowski3 | -0.029 | 0.007 | -4.22 | *** | 0.007 |  |  | -0.012 | 0.003 | -3.95 | *** | 0.006 |  |  |
|  |  |  |  |  |  | 0.19 | 0.11 |  |  |  |  |  | 0.18 | 0.15 |
| Intercept | -0.265 | 0.105 | -2.53 | * |  |  |  | 0.082 | 0.044 | 1.88 | 0.06 |  |  |  |
| Length | 0.009 | 0.004 | 2.46 | * | 0.002 |  |  | -0.009 | 0.001 | -6.14 | *** | 0.014 |  |  |
| Frequency | -0.042 | 0.015 | -2.76 | ** | 0.003 |  |  | 0.019 | 0.006 | 2.94 | ** | 0.003 |  |  |
| Age of Acquisition | 0.026 | 0.004 | 6.69 | *** | 0.017 |  |  | 0.004 | 0.002 | 2.33 | * | 0.002 |  |  |
| Concreteness | 0.294 | 0.023 | 12.71 | *** | 0.061 |  |  | 0.144 | 0.01 | 14.88 | *** | 0.084 |  |  |
| Semantic Diversity | -0.2 | 0.026 | -7.8 | *** | 0.023 |  |  | -0.114 | 0.011 | -10.59 | *** | 0.043 |  |  |
| Euclidean | -0.034 | 0.007 | -5.07 | *** | 0.01 |  |  | -0.013 | 0.003 | -4.61 | *** | 0.008 |  |  |
|  |  |  |  |  |  | 0.2 | 0.12 |  |  |  |  |  | 0.19 | 0.16 |
| Intercept | -0.206 | 0.105 | -1.95 | 0.051 |  |  |  | 0.104 | 0.044 | 2.36 | * |  |  |  |
| Length | 0.009 | 0.004 | 2.69 | ** | 0.003 |  |  | -0.009 | 0.001 | -5.98 | *** | 0.013 |  |  |
| Frequency | -0.04 | 0.015 | -2.69 | ** | 0.003 |  |  | 0.019 | 0.006 | 3.04 | ** | 0.003 |  |  |
| Age of Acquisition | 0.023 | 0.004 | 6 | *** | 0.013 |  |  | 0.003 | 0.002 | 1.75 | 0.081 | 0.001 |  |  |
| Concreteness | 0.291 | 0.023 | 12.61 | *** | 0.059 |  |  | 0.143 | 0.01 | 14.78 | *** | 0.082 |  |  |
| Semantic Diversity | -0.207 | 0.026 | -8.11 | *** | 0.025 |  |  | -0.116 | 0.011 | -10.87 | *** | 0.045 |  |  |
| Sum Strength | -0.032 | 0.005 | -6.53 | *** | 0.016 |  |  | -0.012 | 0.002 | -5.81 | *** | 0.013 |  |  |

*Note*. b represents the unstandardized beta coefficients. SE represents the standard error of the beta coefficients. sr^2^ represents the semi-partial correlation squared. **p*<.05; ***p*<.01; ****p*<.001

**Table S20.** *Regression coefficients from item-level regression analyses predicting Recognition Memory Task hits, false alarms, and hits minus false alarms*

|  | Hits | | | | | | | False Alarms | | | | | | | H– FA | | | | | | |
| --- | --- | --- | --- | --- | --- | --- | --- | --- | --- | --- | --- | --- | --- | --- | --- | --- | --- | --- | --- | --- | --- |
| Predictor | b | SE | t | p | sr2 | R2 | ∆R2 | b | SE | t | p | sr2 | R2 | ∆R2 | b | SE | t | p | sr2 | R2 | ∆R2 |
|  |  |  |  |  |  | 0.28 | 0.10 |  |  |  |  |  | 0.19 | 0.08 |  |  |  |  |  | 0.33 | 0.19 |
| Intercept | 0.719 | 0.002 | 463.18 | *** |  |  |  | 0.218 | 0.002 | 123.24 | 0 |  |  |  | 0.501 | 0.002 | 239.78 | 0 |  |  |  |
| Length | -0.013 | 0.003 | -4.54 | *** | 0.006 |  |  | 0.044 | 0.003 | 13.93 | 0 | 0.061 |  |  | -0.056 | 0.004 | -15.07 | 0 | 0.059 |  |  |
| Frequency | -0.025 | 0.002 | -10.59 | *** | 0.031 |  |  | -0.019 | 0.003 | -6.9 | 0 | 0.015 |  |  | -0.006 | 0.003 | -2.03 | 0.042 | 0.001 |  |  |
| OLD | 0.01 | 0.003 | 3.52 | *** | 0.003 |  |  | -0.046 | 0.003 | -14.42 | 0 | 0.065 |  |  | 0.055 | 0.004 | 14.7 | 0 | 0.056 |  |  |
| Age of Acquisition | 0.012 | 0.002 | 5.2 | *** | 0.007 |  |  | 0.007 | 0.003 | 2.61 | 0.009 | 0.002 |  |  | 0.005 | 0.003 | 1.65 | 0.099 | 0.001 |  |  |
| Concreteness | 0.018 | 0.002 | 8.34 | *** | 0.019 |  |  | -0.005 | 0.002 | -1.83 | 0.067 | 0.001 |  |  | 0.023 | 0.003 | 7.75 | 0 | 0.016 |  |  |
| Semantic Diversity | -0.021 | 0.002 | -11.07 | *** | 0.034 |  |  | 0.022 | 0.002 | 10.09 | 0 | 0.032 |  |  | -0.043 | 0.003 | -16.79 | 0 | 0.073 |  |  |
| Minkowski3 | 0.001 | 0.002 | 0.56 | 0.578 | 0 |  |  | 0.012 | 0.002 | 5.56 | 0 | 0.01 |  |  | -0.011 | 0.003 | -4.25 | 0 | 0.005 |  |  |
|  |  |  |  |  |  | 0.28 | 0.10 |  |  |  |  |  | 0.18 | 0.07 |  |  |  |  |  | 0.33 | 0.19 |
| Intercept | 0.719 | 0.002 | 463.32 | *** |  |  |  | 0.218 | 0.002 | 123.07 | 0 |  |  |  | 0.501 | 0.002 | 239.38 | 0 |  |  |  |
| Length | -0.013 | 0.003 | -4.61 | *** | 0.006 |  |  | 0.044 | 0.003 | 14.03 | *** | 0.062 |  |  | -0.057 | 0.004 | -15.2 | *** | 0.06 |  |  |
| Frequency | -0.026 | 0.002 | -10.83 | *** | 0.032 |  |  | -0.018 | 0.003 | -6.7 | *** | 0.014 |  |  | -0.008 | 0.003 | -2.36 | * | 0.001 |  |  |
| OLD | 0.01 | 0.003 | 3.5 | *** | 0.003 |  |  | -0.046 | 0.003 | -14.45 | *** | 0.066 |  |  | 0.056 | 0.004 | 14.71 | *** | 0.056 |  |  |
| Age of Acquisition | 0.013 | 0.002 | 5.21 | *** | 0.007 |  |  | 0.007 | 0.003 | 2.73 | ** | 0.002 |  |  | 0.005 | 0.003 | 1.55 | 0.121 | 0.001 |  |  |
| Concreteness | 0.019 | 0.002 | 8.68 | *** | 0.021 |  |  | -0.005 | 0.002 | -2.11 | * | 0.001 |  |  | 0.024 | 0.003 | 8.22 | *** | 0.018 |  |  |
| Semantic Diversity | -0.021 | 0.002 | -11.07 | *** | 0.034 |  |  | 0.022 | 0.002 | 10.21 | *** | 0.033 |  |  | -0.043 | 0.003 | -16.87 | *** | 0.074 |  |  |
| Euclidean | 0.003 | 0.002 | 1.38 | 0.169 | 0.001 |  |  | 0.011 | 0.002 | 4.88 | *** | 0.007 |  |  | -0.008 | 0.003 | -3.08 | ** | 0.002 |  |  |
|  |  |  |  |  |  | 0.29 | 0.11 |  |  |  |  |  | 0.18 | 0.07 |  |  |  |  |  | 0.32 | 0.18 |
| Intercept | 0.719 | 0.002 | 464.06 | *** |  |  |  | 0.218 | 0.002 | 122.69 | *** |  |  |  | 0.501 | 0.002 | 238.94 | *** |  |  |  |
| Length | -0.013 | 0.003 | -4.66 | *** | 0.006 |  |  | 0.045 | 0.003 | 14.27 | *** | 0.064 |  |  | -0.058 | 0.004 | -15.43 | *** | 0.062 |  |  |
| Frequency | -0.027 | 0.002 | -11.36 | *** | 0.035 |  |  | -0.016 | 0.003 | -6.06 | *** | 0.012 |  |  | -0.01 | 0.003 | -3.25 | ** | 0.003 |  |  |
| OLD | 0.009 | 0.003 | 3.38 | ** | 0.003 |  |  | -0.046 | 0.003 | -14.43 | *** | 0.066 |  |  | 0.055 | 0.004 | 14.61 | *** | 0.056 |  |  |
| Age of Acquisition | 0.013 | 0.002 | 5.38 | *** | 0.008 |  |  | 0.008 | 0.003 | 2.88 | ** | 0.003 |  |  | 0.005 | 0.003 | 1.53 | 0.126 | 0.001 |  |  |
| Concreteness | 0.02 | 0.002 | 9.48 | *** | 0.025 |  |  | -0.008 | 0.002 | -3.1 | ** | 0.003 |  |  | 0.028 | 0.003 | 9.63 | *** | 0.024 |  |  |
| Semantic Diversity | -0.021 | 0.002 | -10.88 | *** | 0.033 |  |  | 0.023 | 0.002 | 10.33 | *** | 0.034 |  |  | -0.043 | 0.003 | -16.81 | *** | 0.074 |  |  |
| Sum Strength | 0.006 | 0.002 | 3.18 | ** | 0.003 |  |  | 0.006 | 0.002 | 2.8 | ** | 0.002 |  |  | 0 | 0.002 | -0.01 | 0.996 | 0 |  |  |

*Note*. b represents the unstandardized beta coefficients. SE represents the standard error of the beta coefficients. sr^2^ represents the semi-partial correlation squared. **p*<.05; ***p*<.01; ****p*<.001

**Table S21.** *Regression coefficients from item-level regression analyses predicting English Lexicon Project Lexical Decision Task reaction times and error rates including the PCA score*

|  | zRT | | | | | | | Error rate | | | | | | |
| --- | --- | --- | --- | --- | --- | --- | --- | --- | --- | --- | --- | --- | --- | --- |
| Predictor | b | SE | t | p | sr2 | R2 | ∆R2 | b | SE | t | p | sr2 | R2 | ∆R2 |
|  |  |  |  |  |  | 0.53 | 0 |  |  |  |  |  | 0.21 | 0.01 |
| Intercept | -0.261 | 0.003 | -103.3 | *** |  |  |  | 0.06 | 0.001 | 72.18 | *** |  |  |  |
| Length | 0.048 | 0.001 | 36.93 | *** | 0.09 |  |  | -0.009 | 0 | -20.74 | *** | 0.048 |  |  |
| Frequency | -0.129 | 0.005 | -25.03 | *** | 0.041 |  |  | -0.026 | 0.002 | -15.39 | *** | 0.026 |  |  |
| Age of Acquisition | 0.034 | 0.001 | 24.3 | *** | 0.039 |  |  | 0.01 | 0 | 20.8 | *** | 0.048 |  |  |
| Concreteness | -0.003 | 0.003 | -0.99 | 0.321 | 0 |  |  | 0.001 | 0.001 | 1.1 | 0.272 | 0 |  |  |
| Semantic Diversity | -0.07 | 0.01 | -7.08 | *** | 0.003 |  |  | -0.012 | 0.003 | -3.71 | *** | 0.002 |  |  |
| PC1 | -0.014 | 0.003 | -4.78 | *** | 0.001 |  |  | -0.005 | 0.001 | -5.23 | *** | 0.003 |  |  |

*Note*. b represents the unstandardized beta coefficients. SE represents the standard error of the beta coefficients. sr^2^ represents the semi-partial correlation squared. **p*<.05; ***p*<.01; ****p*<.001

**Table S22.** *Regression coefficients from item-level regression analyses predicting English Crowdsourcing Project Word Knowledge Task reaction times and proportion unknown including the PCA score*

|  | zRT | | | | | | | Proportion unknown | | | | | | |
| --- | --- | --- | --- | --- | --- | --- | --- | --- | --- | --- | --- | --- | --- | --- |
| Predictor | b | SE | t | p | sr2 | R2 | ∆R2 | b | SE | t | p | sr2 | R2 | ∆R2 |
|  |  |  |  |  |  | 0.43 | 0.01 |  |  |  |  |  | 0.23 | 0.01 |
| Intercept | -0.533 | 0.001 | -523.19 | *** |  |  |  | 0.012 | 0 | 71.65 | *** |  |  |  |
| Length | 0.012 | 0.001 | 22.31 | *** | 0.039 |  |  | -0.002 | 0 | -20.49 | *** | 0.045 |  |  |
| Frequency | -0.045 | 0.002 | -21.67 | *** | 0.037 |  |  | -0.005 | 0 | -15.3 | *** | 0.025 |  |  |
| Age of Acquisition | 0.012 | 0.001 | 21.98 | *** | 0.038 |  |  | 0.002 | 0 | 21.55 | *** | 0.05 |  |  |
| Concreteness | -0.006 | 0.001 | -4.01 | *** | 0.001 |  |  | 0 | 0 | -0.23 | 0.821 | 0 |  |  |
| Semantic Diversity | -0.023 | 0.004 | -5.92 | *** | 0.003 |  |  | -0.004 | 0.001 | -6.41 | *** | 0.004 |  |  |
| PC1 | -0.013 | 0.001 | -11.57 | *** | 0.011 |  |  | -0.001 | 0 | -7.09 | *** | 0.005 |  |  |

*Note*. b represents the unstandardized beta coefficients. SE represents the standard error of the beta coefficients. sr^2^ represents the semi-partial correlation squared. **p*<.05; ***p*<.01; ****p*<.001

**Table S23.**  *Regression coefficients from item-level regression analyses predicting Calgary Semantic Decision Task reaction times and error rate for concrete words including the PCA score*

|  |  | zRT | | | | | | Error rate | | | | | | |
| --- | --- | --- | --- | --- | --- | --- | --- | --- | --- | --- | --- | --- | --- | --- |
| Predictor | b | SE | t | p | sr2 | R2 | ∆R2 | b | SE | t | p | sr2 | R2 | ∆R2 |
|  |  |  |  |  |  | 0.46 | 0.21 |  |  |  |  |  | 0.32 | 0.23 |
| Intercept | -0.208 | 0.007 | -28.44 | *** |  |  |  | 0.117 | 0.003 | 44.73 | *** |  |  |  |
| Length | 0.016 | 0.004 | 4.12 | *** | 0.005 |  |  | -0.001 | 0.001 | -0.69 | 0.491 | 0 |  |  |
| Frequency | -0.09 | 0.015 | -5.98 | *** | 0.01 |  |  | -0.012 | 0.005 | -2.29 | ** | 0.002 |  |  |
| Age of Acquisition | 0.044 | 0.004 | 12.03 | *** | 0.04 |  |  | 0.007 | 0.001 | 4.99 | *** | 0.009 |  |  |
| Concreteness | -0.491 | 0.019 | -25.37 | *** | 0.177 |  |  | -0.162 | 0.007 | -23.28 | *** | 0.187 |  |  |
| Semantic Diversity | 0.103 | 0.029 | 3.52 | *** | 0.003 |  |  | 0.048 | 0.01 | 4.56 | *** | 0.007 |  |  |
| PC1 | -0.008 | 0.009 | -0.93 | 0.351 | 0 |  |  | 0.001 | 0.003 | 0.21 | 0.833 | 0 |  |  |

*Note*. b represents the unstandardized beta coefficients. SE represents the standard error of the beta coefficients. sr^2^ represents the semi-partial correlation squared. **p*<.05; ***p*<.01; ****p*<.001

**Table S24.**  *Regression coefficients from item-level regression analyses predicting Calgary Semantic Decision Task reaction times and error rate for abstract words including the PCA score*

|  | zRT | | | | | | | Error rate | | | | | | |
| --- | --- | --- | --- | --- | --- | --- | --- | --- | --- | --- | --- | --- | --- | --- |
| Predictor | b | SE | t | p | sr2 | R2 | ∆R2 | b | SE | t | p | sr2 | R2 | ∆R2 |
|  |  |  |  |  |  | 0.2 | 0.12 |  |  |  |  |  | 0.19 | 0.16 |
| Intercept | 0.093 | 0.006 | 14.84 | *** |  |  |  | 0.131 | 0.003 | 49.94 | *** |  |  |  |
| Length | 0.01 | 0.003 | 2.73 | ** | 0.003 |  |  | -0.009 | 0.001 | -5.96 | *** | 0.013 |  |  |
| Frequency | -0.041 | 0.015 | -2.75 | ** | 0.003 |  |  | 0.019 | 0.006 | 3 | ** | 0.003 |  |  |
| Age of Acquisition | 0.022 | 0.004 | 5.73 | *** | 0.012 |  |  | 0.003 | 0.002 | 1.53 | 0.126 | 0.001 |  |  |
| Concreteness | 0.29 | 0.023 | 12.57 | *** | 0.059 |  |  | 0.142 | 0.01 | 14.76 | *** | 0.082 |  |  |
| Semantic Diversity | -0.211 | 0.026 | -8.24 | *** | 0.025 |  |  | -0.118 | 0.011 | -10.99 | *** | 0.045 |  |  |
| PC1 | -0.049 | 0.007 | -6.95 | *** | 0.018 |  |  | -0.018 | 0.003 | -6.11 | *** | 0.014 |  |  |

*Note*. b represents the unstandardized beta coefficients. SE represents the standard error of the beta coefficients. sr^2^ represents the semi-partial correlation squared. **p*<.05; ***p*<.01; ****p*<.001**Table S25.** *Regression coefficients from item-level regression analyses predicting Recognition Memory Task hits, false alarms rates, and hits minus false alarms including the PCA score*

|  | Hits | | | | | | | False Alarms | | | | | | | H - FA | | | | | | |
| --- | --- | --- | --- | --- | --- | --- | --- | --- | --- | --- | --- | --- | --- | --- | --- | --- | --- | --- | --- | --- | --- |
| Predictor | b | SE | t | p | sr2 | R2 | ∆R2 | b | SE | t | p | sr2 | R2 | ∆R2 | b | SE | t | p | sr2 | R2 | ∆R2 |
|  |  |  |  |  |  | 0.29 | 0.11 |  |  |  |  |  | 0.11 | 0 |  |  |  |  |  | 0.27 | 0.13 |
| Length | 0.719 | 0.002 | 463.55 | *** |  |  |  | 0.218 | 0.002 | 117.99 | *** |  |  |  | 0.501 | 0.002 | 229.84 | *** |  |  |  |
| Frequency | -0.005 | 0.002 | -3.3 | ** | 0.003 |  |  | 0.009 | 0.002 | 4.47 | *** | 0.007 |  |  | -0.014 | 0.002 | -6.13 | *** | 0.011 |  |  |
| OLD | -0.027 | 0.002 | -11.63 | *** | 0.037 |  |  | -0.014 | 0.003 | -5.1 | *** | 0.009 |  |  | -0.013 | 0.003 | -3.94 | *** | 0.004 |  |  |
| Age of Acquisition | 0.014 | 0.002 | 5.71 | *** | 0.009 |  |  | 0.005 | 0.003 | 1.81 | 0.071 | 0.001 |  |  | 0.008 | 0.003 | 2.52 | ** | 0.002 |  |  |
| Concreteness | 0.021 | 0.002 | 9.66 | *** | 0.026 |  |  | -0.008 | 0.003 | -3.13 | ** | 0.003 |  |  | 0.029 | 0.003 | 9.51 | *** | 0.025 |  |  |
| Semantic Diversity | -0.021 | 0.002 | -11.12 | *** | 0.034 |  |  | 0.026 | 0.002 | 11.35 | *** | 0.044 |  |  | -0.047 | 0.003 | -17.54 | *** | 0.087 |  |  |
| PC1 | 0.007 | 0.002 | 3.98 | *** | 0.004 |  |  | 0.002 | 0.002 | 1.12 | 0.264 | 0 |  |  | 0.005 | 0.003 | 1.89 | 0.059 | 0.001 |  |  |

*Note*. b represents the unstandardized beta coefficients. SE represents the standard error of the beta coefficients. sr^2^ represents the semi-partial correlation squared. **p*<.05; ***p*<.01; ****p*<.001

**Section S8. Arousal and Dominance**

**Table S26.** *Regression coefficients from item-level regression analyses predicting English Lexicon Project Lexical Decision Task reaction times and error rates for concrete words including arousal and dominance*

|  | zRT | | | | | | | Error rate | | | | | | |
| --- | --- | --- | --- | --- | --- | --- | --- | --- | --- | --- | --- | --- | --- | --- |
| Predictor | b | SE | t | p | sr2 | R2 | ∆R2 | b | SE | t | p | sr2 | R2 | ∆R2 |
| Step1 |  |  |  |  |  | 0.53 | 0.53 |  |  |  |  |  | 0.2 | 0.2 |
| Intercept | -0.261 | 0.003 | -102.67 | *** |  |  |  | 0.06 | 0.001 | 71.84 | *** |  |  |  |
| Length | 0.047 | 0.001 | 36.51 | *** | 0.089 |  |  | -0.009 | 0 | -21.97 | *** | 0.054 |  |  |
| Frequency | -0.146 | 0.005 | -30.42 | *** | 0.062 |  |  | -0.031 | 0.002 | -19.5 | *** | 0.042 |  |  |
| Age of Acquisition | 0.034 | 0.001 | 26.27 | *** | 0.046 |  |  | 0.009 | 0 | 21.58 | *** | 0.052 |  |  |
| Step2 |  |  |  |  |  | 0.54 | 0.01 |  |  |  |  |  | 0.22 | 0.01 |
| Intercept | -0.261 | 0.003 | -104.11 | *** |  |  |  | 0.06 | 0.001 | 72.35 | *** |  |  |  |
| Length | 0.05 | 0.001 | 38.02 | *** | 0.093 |  |  | -0.009 | 0 | -19.87 | *** | 0.043 |  |  |
| Frequency | -0.122 | 0.005 | -23.62 | *** | 0.036 |  |  | -0.025 | 0.002 | -14.33 | *** | 0.023 |  |  |
| Age of Acquisition | 0.034 | 0.001 | 24.12 | *** | 0.038 |  |  | 0.01 | 0 | 20.9 | *** | 0.048 |  |  |
| Concreteness | -0.007 | 0.003 | -2.05 | ** | 0 |  |  | 0 | 0.001 | 0.29 | 0.769 | 0 |  |  |
| Valence Extremity | 0.002 | 0.004 | 0.45 | 0.655 | 0 |  |  | 0 | 0.001 | -0.27 | 0.79 | 0 |  |  |
| Arousal | 0.011 | 0.003 | 3.42 | 0.001 | 0.001 |  |  | 0.002 | 0.001 | 1.9 | 0.057 | 0 |  |  |
| Dominance | -0.014 | 0.003 | -4.67 | *** | 0.001 |  |  | -0.001 | 0.001 | -0.88 | 0.377 | 0 |  |  |
| Semantic Diversity | -0.056 | 0.01 | -5.64 | *** | 0.002 |  |  | -0.009 | 0.003 | -2.75 | ** | 0.001 |  |  |
| Motivation | -0.026 | 0.003 | -7.93 | *** | 0.004 |  |  | -0.008 | 0.001 | -6.98 | *** | 0.005 |  |  |

*Note*. b represents the unstandardized beta coefficients. SE represents the standard error of the beta coefficients. sr^2^ represents the semi-partial correlation squared. **p*<.05; ***p*<.01; ****p*<.001

**Table S27.** *Regression coefficients from item-level regression analyses predicting English Crowdsourcing Project Word Knowledge Task reaction times and proportion unknown including arousal and dominance*

|  | zRT | | | | | | | Proportion Unknown | | | | | | |
| --- | --- | --- | --- | --- | --- | --- | --- | --- | --- | --- | --- | --- | --- | --- |
| Predictor | b | SE | t | p | sr2 | R2 | ∆R2 | b | SE | t | p | sr2 | R2 | ∆R2 |
| Step1 |  |  |  |  |  | 0.42 | 0.42 |  |  |  |  |  | 0.22 | 0.22 |
| Intercept | -0.533 | 0.001 | -517.16 | *** |  |  |  | 0.012 | 0 | 71.05 | *** |  |  |  |
| Length | 0.011 | 0.001 | 20.37 | *** | 0.034 |  |  | -0.002 | 0 | -21.96 | *** | 0.052 |  |  |
| Frequency | -0.054 | 0.002 | -27.73 | *** | 0.062 |  |  | -0.007 | 0 | -20.54 | *** | 0.046 |  |  |
| Age of Acquisition | 0.013 | 0.001 | 24.7 | *** | 0.049 |  |  | 0.002 | 0 | 22.7 | *** | 0.056 |  |  |
| Step2 |  |  |  |  |  | 0.43 | 0.02 |  |  |  |  |  | 0.23 | 0.02 |
| Intercept | -0.533 | 0.001 | -524.51 | *** |  |  |  | 0.012 | 0 | 71.74 | *** |  |  |  |
| Length | 0.012 | 0.001 | 22.68 | *** | 0.041 |  |  | -0.002 | 0 | -19.87 | *** | 0.042 |  |  |
| Frequency | -0.044 | 0.002 | -20.86 | *** | 0.034 |  |  | -0.005 | 0 | -14.45 | *** | 0.022 |  |  |
| Age of Acquisition | 0.012 | 0.001 | 21.34 | *** | 0.036 |  |  | 0.002 | 0 | 21.83 | *** | 0.051 |  |  |
| Concreteness | -0.006 | 0.001 | -4.42 | *** | 0.002 |  |  | 0 | 0 | -0.83 | 0.404 | 0 |  |  |
| Valence Extremity | -0.008 | 0.001 | -5.41 | *** | 0.002 |  |  | 0 | 0 | -0.94 | 0.346 | 0 |  |  |
| Arousal | 0 | 0.001 | -0.2 | 0.843 | 0 |  |  | 0 | 0 | -1.58 | 0.115 | 0 |  |  |
| Dominance | -0.005 | 0.001 | -4.34 | *** | 0.001 |  |  | 0 | 0 | 0.7 | 0.485 | 0 |  |  |
| Semantic Diversity | -0.021 | 0.004 | -5.29 | *** | 0.002 |  |  | -0.004 | 0.001 | -5.9 | *** | 0.004 |  |  |
| Motivation | -0.01 | 0.001 | -7.79 | *** | 0.005 |  |  | -0.001 | 0 | -6.53 | *** | 0.005 |  |  |

*Note*. b represents the unstandardized beta coefficients. SE represents the standard error of the beta coefficients. sr^2^ represents the semi-partial correlation squared. **p*<.05; ***p*<.01; ****p*<.001

**Table S28.** *Regression coefficients from item-level regression analyses predicting Calgary Semantic Decision Task reaction times and error rates for concrete words including arousal and dominance*

|  | zRT | | | | | | | Error rate | | | | | | |
| --- | --- | --- | --- | --- | --- | --- | --- | --- | --- | --- | --- | --- | --- | --- |
| Predictor | b | SE | t | p | sr2 | R2 | ∆R2 | b | SE | t | p | sr2 | R2 | ∆R2 |
| Step1 |  |  |  |  |  | 0.25 | 0.25 |  |  |  |  |  | 0.09 | 0.09 |
| Intercept | -0.208 | 0.009 | -24.14 | *** |  |  |  | 0.117 | 0.003 | 38.56 | *** |  |  |  |
| Length | 0.03 | 0.009 | 3.26 | ** | 0.004 |  |  | -0.002 | 0.003 | -0.74 | 0.457 | 0 |  |  |
| Frequency | -0.036 | 0.01 | -3.57 | *** | 0.005 |  |  | 0.001 | 0.004 | 0.38 | 0.702 | 0 |  |  |
| Age of Acquisition | 0.193 | 0.01 | 19.4 | *** | 0.143 |  |  | 0.044 | 0.004 | 12.42 | *** | 0.072 |  |  |
| Step2 |  |  |  |  |  | 0.46 | 0.21 |  |  |  |  |  | 0.32 | 0.24 |
| Intercept | -0.208 | 0.007 | -28.46 | *** |  |  |  | 0.117 | 0.003 | 44.79 | *** |  |  |  |
| Length | 0.034 | 0.008 | 4.23 | *** | 0.005 |  |  | -0.002 | 0.003 | -0.76 | 0.448 | 0 |  |  |
| Frequency | -0.054 | 0.009 | -5.66 | *** | 0.009 |  |  | -0.008 | 0.003 | -2.29 | * | 0.002 |  |  |
| Age of Acquisition | 0.107 | 0.009 | 11.46 | *** | 0.036 |  |  | 0.014 | 0.003 | 4.33 | *** | 0.006 |  |  |
| Concreteness | -0.213 | 0.009 | -24.59 | *** | 0.166 |  |  | -0.069 | 0.003 | -22.17 | *** | 0.169 |  |  |
| Valence Extremity | -0.003 | 0.008 | -0.33 | 0.738 | 0 |  |  | -0.002 | 0.003 | -0.76 | 0.446 | 0 |  |  |
| Arousal | -0.007 | 0.009 | -0.86 | 0.389 | 0 |  |  | -0.003 | 0.003 | -0.89 | 0.374 | 0 |  |  |
| Dominance | -0.018 | 0.008 | -2.22 | * | 0.001 |  |  | -0.008 | 0.003 | -2.77 | ** | 0.003 |  |  |
| Semantic Diversity | 0.028 | 0.008 | 3.49 | ** | 0.003 |  |  | 0.013 | 0.003 | 4.34 | *** | 0.007 |  |  |
| Motivation | -0.007 | 0.009 | -0.76 | 0.449 | 0 |  |  | 0.004 | 0.003 | 1.07 | 0.285 | 0 |  |  |

*Note*. b represents the unstandardized beta coefficients. SE represents the standard error of the beta coefficients. sr^2^ represents the semi-partial correlation squared. **p*<.05; ***p*<.01; ****p*<.001

**Table S29.** *Regression coefficients from item-level regression analyses predicting Calgary Semantic Decision Task reaction times and error rates for abstract words including arousal and dominance*

|  |  | zRT | | | | | | | | Error rate | | | | | | | |
| --- | --- | --- | --- | --- | --- | --- | --- | --- | --- | --- | --- | --- | --- | --- | --- | --- | --- |
| Predictor | | | b | SE | t | p | sr2 | R2 | ∆R2 | | b | SE | t | p | sr2 | R2 | ∆R2 |
| Step1 | | |  |  |  |  |  | 0.08 | 0.08 | |  |  |  |  |  | 0.03 | 0.03 |
| Intercept | | | 0.056 | 0.074 | 0.75 | 0.451 |  |  |  | | 0.225 | 0.032 | 7.08 | *** |  |  |  |
| Length | | | -0.001 | 0.004 | -0.18 | 0.855 | 0 |  |  | | -0.013 | 0.002 | -8.51 | *** | 0.033 |  |  |
| Frequency | | | -0.101 | 0.015 | -6.6 | *** | 0.019 |  |  | | -0.01 | 0.007 | -1.54 | 0.124 | 0.001 |  |  |
| Age of Acquisition | | | 0.024 | 0.004 | 5.95 | *** | 0.015 |  |  | | 0.003 | 0.002 | 1.71 | 0.087 | 0.001 |  |  |
| Step2 | | |  |  |  |  |  | 0.2 | 0.12 | |  |  |  |  |  | 0.19 | 0.16 |
| Intercept | | | -0.143 | 0.118 | -1.21 | 0.226 |  |  |  | | 0.127 | 0.049 | 2.57 | * |  |  |  |
| Length | | | 0.009 | 0.003 | 2.62 | 0.009 | 0.003 |  |  | | -0.009 | 0.001 | -6.05 | *** | 0.014 |  |  |
| Frequency | | | -0.047 | 0.015 | -3.12 | ** | 0.004 |  |  | | 0.017 | 0.006 | 2.68 | ** | 0.003 |  |  |
| Age of Acquisition | | | 0.021 | 0.004 | 5.13 | *** | 0.01 |  |  | | 0.002 | 0.002 | 1.14 | 0.252 | 0 |  |  |
| Concreteness | | | 0.289 | 0.023 | 12.54 | *** | 0.059 |  |  | | 0.142 | 0.01 | 14.71 | *** | 0.081 |  |  |
| Valence Extremity | | | -0.054 | 0.009 | -5.89 | *** | 0.013 |  |  | | -0.019 | 0.004 | -4.92 | *** | 0.009 |  |  |
| Arousal | | | -0.009 | 0.008 | -1.15 | 0.252 | 0 |  |  | | -0.003 | 0.003 | -0.89 | 0.376 | 0 |  |  |
| Dominance | | | 0.002 | 0.007 | 0.25 | 0.804 | 0 |  |  | | 0 | 0.003 | -0.02 | 0.984 | 0 |  |  |
| Semantic Diversity | | | -0.223 | 0.026 | -8.6 | *** | 0.028 |  |  | | -0.121 | 0.011 | -11.16 | *** | 0.047 |  |  |
| Motivation | | | -0.015 | 0.009 | -1.71 | 0.088 | 0.001 |  |  | | -0.006 | 0.004 | -1.71 | 0.087 | 0.001 |  |  |

*Note*. b represents the unstandardized beta coefficients. SE represents the standard error of the beta coefficients. sr^2^ represents the semi-partial correlation squared. **p*<.05; ***p*<.01; ****p*<.001

**Table S30.** *Regression coefficients from item-level regression analyses predicting Recognition Memory Task hits and false alarms rates including arousal and dominance*

|  | Hits | | | | | | | False alarms | | | | | | | |
| --- | --- | --- | --- | --- | --- | --- | --- | --- | --- | --- | --- | --- | --- | --- | --- |
| Predictor | b | SE | t | p | sr2 | R2 | ∆R2 | b | SE | t | p | sr2 | R2 | R2 | ∆R2 |
| Step1 |  |  |  |  |  | 0.18 | 0.18 |  |  |  |  |  | 0.11 | 0.14 | 0.14 |
| Intercept | 0.719 | 0.002 | 433.3 | *** |  |  |  | 0.218 | 0.002 | 117.84 | *** |  |  |  |  |
| Length | -0.016 | 0.003 | -5.45 | *** | 0.009 |  |  | 0.049 | 0.003 | 15.02 | *** | 0.077 |  |  |  |
| Frequency | -0.041 | 0.002 | -18.09 | *** | 0.103 |  |  | 0 | 0.003 | -0.08 | 0.939 | 0 |  |  |  |
| OLD | 0.013 | 0.003 | 4.5 | *** | 0.006 |  |  | -0.049 | 0.003 | -14.93 | *** | 0.076 |  |  |  |
| Age of Acquisition | -0.002 | 0.002 | -1 | 0.317 | 0 |  |  | 0.018 | 0.003 | 7.12 | *** | 0.017 |  |  |  |
| Step2 |  |  |  |  |  | 0.31 | 0.13 |  |  |  |  |  | 0.19 | 0.35 | 0.21 |
| Intercept | 0.719 | 0.002 | 471.49 | *** |  |  |  | 0.218 | 0.002 | 123.84 | *** |  |  |  |  |
| Length | -0.01 | 0.003 | -3.68 | *** | 0.004 |  |  | 0.043 | 0.003 | 13.6 | *** | 0.057 |  |  |  |
| Frequency | -0.025 | 0.002 | -10.73 | *** | 0.031 |  |  | -0.018 | 0.003 | -6.77 | *** | 0.014 |  |  |  |
| OLD | 0.007 | 0.003 | 2.68 | ** | 0.002 |  |  | -0.045 | 0.003 | -13.96 | *** | 0.06 |  |  |  |
| Age of Acquisition | 0.013 | 0.002 | 5.59 | *** | 0.008 |  |  | 0.004 | 0.003 | 1.56 | 0.118 | 0.001 |  |  |  |
| Concreteness | 0.018 | 0.002 | 8.49 | *** | 0.019 |  |  | -0.005 | 0.002 | -1.88 | 0.06 | 0.001 |  |  |  |
| Valence Extremity | 0.006 | 0.002 | 3.67 | *** | 0.004 |  |  | -0.009 | 0.002 | -4.5 | *** | 0.006 |  |  |  |
| Arousal | 0.012 | 0.002 | 6.81 | *** | 0.012 |  |  | 0.001 | 0.002 | 0.63 | 0.528 | 0 |  |  |  |
| Dominance | -0.003 | 0.002 | -1.61 | 0.108 | 0.001 |  |  | -0.004 | 0.002 | -1.79 | 0.074 | 0.001 |  |  |  |
| Semantic Diversity | -0.018 | 0.002 | -9.17 | *** | 0.022 |  |  | 0.02 | 0.002 | 9.17 | *** | 0.026 |  |  |  |
| Motivation | -0.007 | 0.002 | -3.18 | ** | 0.003 |  |  | 0.016 | 0.003 | 6.59 | *** | 0.013 |  |  |  |

*Note*. b represents the unstandardized beta coefficients. SE represents the standard error of the beta coefficients. sr^2^ represents the semi-partial correlation squared. **p*<.05; ***p*<.01; ****p*<.001

**Table S31.** *Regression coefficients from item-level regression analyses predicting the Recognition Memory Task hits minus false alarms including arousal and dominance*

|  | H - FA | | | | | | |
| --- | --- | --- | --- | --- | --- | --- | --- |
| Predictor | b | SE | t | p | sr2 | R2 | ∆R2 |
| Step1 |  |  |  |  |  | 0.14 | 0.14 |
| Intercept | 0.501 | 0.002 | 211.92 | *** |  |  |  |
| Length | -0.065 | 0.004 | -15.49 | *** | 0.079 |  |  |
| Frequency | -0.041 | 0.003 | -12.63 | *** | 0.053 |  |  |
| OLD | 0.063 | 0.004 | 14.74 | *** | 0.072 |  |  |
| Age of Acquisition | -0.02 | 0.003 | -6.27 | *** | 0.013 |  |  |
| Step2 |  |  |  |  |  | 0.35 | 0.21 |
| Intercept | 0.501 | 0.002 | 244.17 | *** |  |  |  |
| Length | -0.053 | 0.004 | -14.32 | *** | 0.051 |  |  |
| Frequency | -0.007 | 0.003 | -2.16 | * | 0.001 |  |  |
| OLD | 0.052 | 0.004 | 13.85 | *** | 0.048 |  |  |
| Age of Acquisition | 0.009 | 0.003 | 2.82 | ** | 0.002 |  |  |
| Concreteness | 0.023 | 0.003 | 7.92 | *** | 0.016 |  |  |
| Valence Extremity | 0.015 | 0.002 | 6.57 | *** | 0.011 |  |  |
| Arousal | 0.011 | 0.002 | 4.52 | *** | 0.005 |  |  |
| Dominance | 0.001 | 0.002 | 0.45 | 0.653 | 0 |  |  |
| Semantic Diversity | -0.038 | 0.003 | -14.72 | *** | 0.054 |  |  |
| Motivation | -0.023 | 0.003 | -7.99 | *** | 0.016 |  |  |

*Note*. b represents the unstandardized beta coefficients. SE represents the standard error of the beta coefficients. sr^2^ represents the semi-partial correlation squared. **p*<.05; ***p*<.01; ****p*<.001
